# Evidence of Differential Allelic Effects between Adolescents and Adults for Plasma High-Density Lipoprotein

**DOI:** 10.1371/journal.pone.0035605

**Published:** 2012-04-18

**Authors:** Rita P. S. Middelberg, Andrew C. Heath, Pamela A. F. Madden, Grant W. Montgomery, Nicholas G. Martin, John B. Whitfield

**Affiliations:** 1 Genetic Epidemiology, Queensland Institute of Medical Research, Brisbane, Australia; 2 Department of Medicine, Prince Charles Hospital, Queensland, Australia; 3 Department of Psychiatry, Washington University School of Medicine and Midwest Alcoholism Research Center, St. Louis, Missouri, United States of America; 4 Molecular Epidemology, Queensland Institute of Medical Research, Brisbane, Australia; Leibniz-Institute for Arteriosclerosis Research at the University Muenster, Germany

## Abstract

A recent meta-analysis of genome-wide association (GWA) studies identified 95 loci that influence lipid traits in the adult population and found that collectively these explained about 25–30% of heritability for each trait. Little is known about how these loci affect lipid levels in early life, but there is evidence that genetic effects on HDL- and LDL-cholesterol (HDL-C, LDL-C) and triglycerides vary with age. We studied Australian adults (N = 10,151) and adolescents (N = 2,363) who participated in twin and family studies and for whom we have lipid phenotypes and genotype information for 91 of the 95 genetic variants. Heterogeneity tests between effect sizes in adult and adolescent cohorts showed an excess of heterogeneity for HDL-C (p_Het_<0.05 at 5 out of 37 loci), but no more than expected by chance for LDL-C (1 out of 14 loci), or trigycerides (0 out 24). There were 2 (out of 5) with opposite direction of effect in adolescents compared to adults for HDL-C, but none for LDL-C. The biggest difference in effect size was for LDL-C at rs6511720 near *LDLR*, adolescents (0.021±0.033 mmol/L) and adults (0.157±0.023 mmol/L), p_Het_ = 0.013; followed by *ZNF664* (p_Het_ = 0.018) and *PABPC4* (p_Het_ = 0.034) for HDL-C. Our findings suggest that some of the previously identified variants associate differently with lipid traits in adolescents compared to adults, either because of developmental changes or because of greater interactions with environmental differences in adults.

## Introduction

Plasma lipids are important and much-studied risk factors for cardiovascular disease. For clinical use and in epidemiological studies the main focus is on concentrations of low-density lipoprotein cholesterol (LDL-C), high-density lipoprotein cholesterol (HDL-C) and triglycerides. These are known to be substantially influenced by genetic variation, with heritabilities of 40%–60% in adults and even higher (70%–80%) in adolescents. Many novel loci affecting lipid risk factors for cardiovascular disease have been discovered through meta-analysis of genome-wide association (GWAS) data [1], [2]. A recent and even larger meta-analysis [3] identified 95 susceptibility loci that influence HDL-C, LDL-C or triglycerides in adults. The significant common variants explained 10%–12% of the total variation in lipid levels, or around 25% of the heritability. Such results help to define pathways between gene polymorphisms and their clinical effects, allow some degree of risk stratification, and may identify novel targets for risk-reducing drugs.

However, it is known that mean values for plasma lipids change with age, and there is some evidence that the gene variants which contribute to differences between people also change across the lifespan. Longitudinal studies of twins across adolescence or early adulthood [4], [5], [6] and cross-generational comparisons of parents and offspring [7] support this concept. A GWAS based on longitudinal cardiovascular risk factor data, with 525 genotyped participants and repeated measurements covering the age range 4 to 48 years [8], showed suggestive but non-replicated SNP-by-age interactions for two loci with LDL-C and for five (two at genome-wide significance) for triglycerides.

Comparatively little is known about genetic loci affecting lipid levels in early life, despite higher heritabilities in adolescents than in adults. Practically all the published GWAS data are from adults, often from the older adults at highest risk of cardiovascular disease. While this is logical, atherosclerosis may start early in life, and the question of whether the SNPs and genes already identified affect lipid variation in childhood or adolescence is a relevant one. Practical benefits could ensue if SNPs which affect adult levels of risk factors, but not childhood levels, could be identified; intervention based on a genetic profile could potentially be started well before the risk becomes evident from conventional (phenotypic) risk factor assessments [8].

We have analysed lipid GWAS data from adolescents, concentrating on the genes and SNPs shown to be important in adults and testing for heterogeneity of allelic effects between adult (N = 10,151) and adolescent (N = 2363) cohorts drawn from the Australian population. The opposite approach, of starting with polymorphisms significantly affecting lipids in adolescence and comparing them against adult results, is limited by the comparative lack of data on young subjects. We first tested for age heterogeneity within the adolescents and the adults separately. After showing a lack of significant heterogeneity by age within these two groups, allelic association tests on each cohort were performed separately. Heterogeneity tests on each lipid trait at 91 loci with genotyping were conducted to determine whether there is any difference in the effect of these significant reported SNPs on plasma lipid concentrations between adolescents and adults.

## Methods

### Subjects

Lipid traits (HDL-C, LDL-C, triglycerides) were measured in serum samples from twins and their families, and genome-wide SNP markers were genotyped. The study participants consist of:

1. Adolescent twins and their non-twin siblings living in south-east Queensland (Australia) who had participated in the Brisbane Longitudinal Twin study [9], [10], [11], [12]. Full details are described in Middelberg et al. [5]. A total of 2363 participants (1196 females and 1167 males; mean age of 14.5 years) were genotyped.
2. Adults (twins and their family members) who participated in studies of: (i) alcohol and nicotine use and dependence and metabolic risk for alcoholic liver disease [13] (n = 6924); (ii) Anxiety and Depression [14] (n = 1213); (iii) Endometriosis [15] (n = 845); and (iv) Pre-1982 twin studies; Alcohol Challenge and follow-up and vitamin C [16], [17], [18], [19] (n = 1171). A total of 10,151 individuals (6257 females and 3894 males; mean age of 45.7 years) were genotyped in these community-recruited particpants.

There was no overlap between participation in the adolescent and adult studies. However, some adults participated in more than one study and many of the adolescents had phenotypic measurements on more than one occasion. Combining all these studies, each individual was first categorised into age groups. For the adolescents these were 1 (age 12–13.99 years), 2 (age 14–15.99 years) or 3 (16–17.99 years). Adult groups were 4 (18–29.99 years), 5 (30–44.99 years), 6 (45–59.99 years) or 7 (age 60 years and over). The present study includes 20,634 individuals with HDL-C, LDL-C and triglycerides measurements and 12,514 from 5,424 families (2,363 individuals from 1,024 adolescent families; 10,151 individual from 4,400 adult families) who had both genotype and phenotype data. All participants (and, for subjects aged <18 years, their parents) gave informed written consent and all studies were approved by the Human Research Ethics Committee of the Queensland Institute of Medical Research.

### Laboratory measurements

Serum was separated from the blood samples and stored at −70°C until analyzed. Serum cholesterol, HDL cholesterol and triglycerides, were measured using Roche methods on a Roche 917 or Modular P analyzer (Roche Diagnostics, Basel, Switzerland). LDL-cholesterol was calculated using the Friedewald equation.

### Genotyping

DNA was extracted from blood samples using standard methods and genotyped with Illumina 610K, 317K or 370K chips at CIDR or deCODE Genetics. Data cleaning for SNP genotypes included checking the expected relationships between individual family members and resolving Mendelian errors [20]. Imputed genotypes for SNPs were generated using *MACH 1.0* (http://www.sph.umich.edu/csg/abecasis/mach/index.html) [21], [22] program with the HapMap CEU I+II (release 22, build 36) reference panel. Any imputed SNP which had 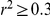 was included in the genotype data.

### Statistical Analysis

Distributions of lipid variables were examined and triglyceride was log-transformed. Individuals whose results for any trait were more than five standard deviations from the mean were excluded for that trait. Before association analysis, the variables were adjusted for the effects of age, squared age (age^2^), sex, sex×age and sex×age^2^ and standardized residuals were obtained. All data pre-processing and descriptive analyses were done using *STATA* version 7.0 [23] and *SPSS* version 17.0.2 (Mar 11, 2009). SNP family-based association analysis was performed using “*fastassoc*” and “*inverseNormal*” options in *MERLIN 1.1.2*. [24]. To examine whether there are differences in effect sizes within the adolescent or adult cohorts, we first obtained the effect size, direction and standard error for each SNP identified in Teslovich's study [3], in each age category from family-based association analyses. Then, heterogeneity within the children/adolescents (that is, age categories 1–3) and within the adults (age categories 4–7) were tested separately. As no evidence for heterogeneity within each group was found, a heterogeneity test contrasting effect sizes between adolescents and adults was performed. For the initial comparisons of the different age-groups within adolescence or within adulthood, no adjustment was made for multiple observations from the same person at different ages. This is a preliminary check for heterogeneity within adolescent or adult cohorts. For the subsequent analysis (that is, the adolescent versus adult heterogeneity tests), where there were multiple measurements of the same trait in an individual, an average of the values was used. For the final heterogeneity test, estimates of the effect sizes and standard errors were calculated using MERLIN (allowing for familial relationships) and compared between adolescent and adult cohorts. Heterogeneity tests were performed using *METAL 1.1.2* [25].

## Results

Means and standard deviations of lipid traits for males and females in adolescent and adult cohorts are listed in Table 1. Generally, the lipid traits have lower means in the adolescents than the adults, as expected. Out of the 102 SNPs (at 95 loci) identified by the Global Lipids Consortium [3], only 98 SNPs (at 91 loci) were examined in our study; four SNPs were excluded due to poor imputation quality score (R^2^<0.3) or violation of Hardy-Weinberg equilibrium (at P<10^−6^). Most SNPs (23/24 for HDL-C, 12/14 for LDL-C and 35/36 for triglycerides) associated with lipid concentrations (as the lead trait) in our adult data were directionally consistent with the Global Lipids Consortium meta-analysis. The correlations between effect sizes reported by the Global Lipids Consortium for the SNPs they found significant and our effect sizes were 0.94 for HDL-C, 0.98 for LDL-C and 0.95 for triglycerides. In adolescents, 41 out of the 98 SNPs were significantly (p<0.05) associated with at least one of the lipid traits (Table S1). The correlations of effect sizes between our adolescent and the Global Lipids Consortium results were 0.84 for HDL-C, 0.84 for LDL-C and 0.91 for triglycerides.

**Table 1:** Descriptive characteristics of genotyped adolescents and adults stratified by sex.

Trait	Adolescents (n = 2363)						Adults (n = 10151)					
	Male (n = 1167)			Female (n = 1196)			Male (n = 3894)			Female (n = 6257)		
	N	Mean	SD	N	Mean	SD	N	Mean	SD	N	Mean	SD
**Age (** ***years*** **)**	1167	14.46	1.54	1196	14.62	1.68	3894	46.50	12.73	6257	45.21	13.10
**HDL-C**(mmol/L)	1164	1.34	0.29	1196	1.43	0.29	3875	1.33	0.35	6224	1.64	0.41
**LDL-C**(mmol/L)	1160	2.38	0.67	1196	2.47	0.61	3656	3.41	0.91	6122	3.21	0.92
**Triglycerides** (mmol/L)	1164	1.26	0.63	1196	1.15	0.50	3892	2.21	1.40	6248	1.61	0.97

Heterogeneity tests generally showed no significant difference (at p<0.05) in effect sizes between the three age groups (within adolescents) except for *LDLR* and *HFE* on HDL-C (p_Het_ = 0.049 and 0.024), *ERGIC3* on LDL-C (p_Het_ = 0.014) and *SCARB1* on LTG (p_Het_ = 0.031) (Tables S2, S3, S4). Similarly there was no significant heterogeneity in effect size between the four age groups within the adults except for *UBE2L3* and *PINX1* on HDL-C (p_Het_ = 0.020 and 0.031), *COBLL1*, *APOE*, *ANGPTL3*, *HFE*, *MAFB*, *HMGCR* and *ZNF664* on LDL-C (p_Het_ = 0.455, 0.017, 0.032, 0.028, 0.040, 0.012, 0.046) and *FLJ36070*, *GALNT2*, *SLC39A8*, PDE3A and *PINX1* on triglycerides (p_Het_ = 0.015, 0.021, 0.009, 0.024, 0.013) (Tables S2, S3, S4). Hence, no significant heterogeneity in effect sizes was detected within the adolescents or adults after Bonferroni correction for multiple testing.

**Figure 1:**
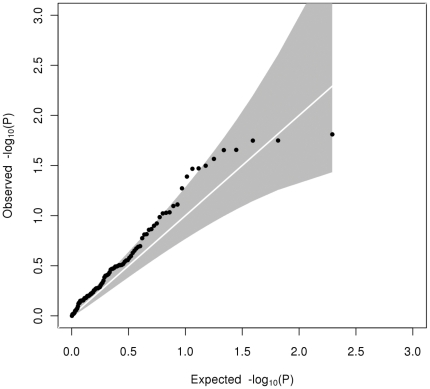
Quantile-quantile plot of observed against expected heterogeneity P-value for allelic associations with HDL-C. The grey shaded area represents the 95% confidence interval.

**Figure 2:**
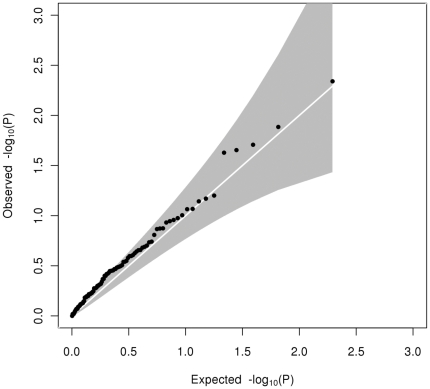
Quantile-quantile plot of observed against expected heterogeneity P-value for allelic associations with LDL-C. The grey shaded area represents the 95% confidence interval.

**Figure 3:**
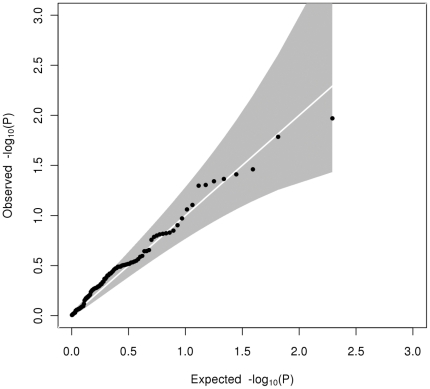
Quantile-quantile plot of observed against expected heterogeneity P-value for allelic associations with triglycerides. The grey shaded area represents the 95% confidence interval.

Heterogeneity testing between the adolescent and adult cohorts showed there were 5 (out of 37), 1 (out of 14) and 0 (out of 24) variants showing significant heterogeneity (p<0.05) in effect size for HDL-C (most significant p_Het_ = 0.016 at *MC4R*), LDL-C (significant p_Het_ = 0.013 at *LDLR*) and triglycerides respectively (Table 2 & Tables S5, S6, S7). For HDL-C, but not for LDL-C or triglycerides, there was an excess of significantly heterogeneous associations among these 98 SNPs over expectation (Figures 1, 2, 3). The biggest difference in effect size was the G-allele of rs6511720 (*LDLR* gene) showing a 7-fold difference in association with LDL-C (effect size of 0.033±0.051 in SD unit (equivalent to 0.021±0.033 mmol/L) in adolescents and 0.174±0.051 in SD (equivalent to 0.157±0.023 mmol/L) in adults). Some SNPs in HDL-C were seen to have opposite direction of effect in adolescents compared to adults. The biggest difference in effect direction was for the A-allele of rs4660293 in *PABPC4* (−0.029±0.038 in SD units (equivalent to −0.008±0.011 mmol/L) in adolescents; 0.061±0.019 in SD unit (equivalent to 0.024±0.007 mmol/L) in adults). There were also some SNPs showing an increase or decrease in effect size without change in direction from adolescents to adults. In addition to rs7134594 (gene *MVK*) with HDL-C, for which the effect size of the T-allele was almost 6-fold higher in adolescents than in adults, rs12967135 (*MC4R* gene) also showed a 3-fold higher in effect size of the G-allele in adolescents compared to adults. In addition, one variant showed a p-value of less than 0.05 in heterogeneity between adolescents and adults in more than one trait. The effect size of the G-allele of rs6511720 in *LDLR* was differentially associated with both HDL-C (0.102±0.051 in SD (equivalent to 0.029±0.015 mmol/L) in adolescents; −0.019±0.024 in SD (equivalent to −0.007±0.009 mmol/L) in adults; p_Het_ = 0.032) and LDL-C (0.033±0.051 in SD (equivalent to 0.021±0.033 mmol/L) in adolescents; 0.174±0.025 in SD (equivalent to 0.157±0.023 mmol/L) in adults; p_Het_ = 0.013).

**Table 2:** Loci exhibiting adult-adolescent heterogeneity at p<0.05. The locus/phenotype combinations tested were based on the significant associations reported for adults by Teslovich et al.

						Adolescents			Adults			Adolescents+Adults				
Trait	Locus	Chr	SNP	Allele/MAF	Ref Allele	Beta	SE	P-value	Beta	SE	P-value	Beta	SE	P-value	Direction	Het p-value
HDL	PABPC4	1	rs4660293	A/G/0.25	A	−0.029	0.038	0.450	0.061	0.019	0.0012	0.043	0.017	0.011	−+	0.034
	ZNF664	12	rs4765127	G/T/0.34	G	0.009	0.034	0.800	−0.080	0.016	8.00×10^−07^	−0.064	0.015	1.03×10^−05^	−+	0.018
	MVK	12	rs7134594	T/C/0.47	T	0.085	0.032	0.008	0.010	0.015	0.510	0.024	0.014	0.083	++	0.034
	CETP	16	rs3764261	C/A/0.32	C	−0.330	0.035	9.80×10^−22^	−0.245	0.016	1.20×10^−50^	−0.260	0.015	3.09×10^−71^	−−	0.027
	MC4R	18	rs12967135	G/A/0.24	G	0.136	0.039	0.0005	0.032	0.018	0.077	0.050	0.016	0.0021	++	0.016
LDL	LDLR	19	rs6511720	G/T/0.11	G	0.033	0.051	0.520	0.174	0.025	2.70×10^−12^	0.147	0.022	6.39×10^−11^	++	0.013

“Allele/MAF” listed are: major allele, minor allele frequency (MAF). Numbers in “Beta” columns are in SD units, modelled as additive effect of the reference allele.

We also generated a Q-Q plot of the heterogeneity p-values across all (≈2.5 million) SNPs genome-wide. These did not show genome-wide significant (p<5×10^−8^) heterogeneity of allelic effects between adolescents and adults (Figures S1, S2, S3), and there was no evidence for an excess of suggestive associations above those which would be expected by chance.

## Discussion

Our study examined and compared allelic effects in 2,363 adolescents and 10,151 adults at 91 previously identified loci known to influence high- or low-density lipoprotein cholesterol, or triglycerides, in adults. The allelic associations found in our data are consistent with previous reports, given the smaller number of subjects available. We found a high correlation between effect sizes reported by the Global Lipids Consortium for the SNPs they found significant [3] and the effect sizes found in our adult (r^2^>0.90) and adolescent (r^2^>0.80) results. All SNPs giving significant associations at p<5×10^−8^ in our adolescent or adult data had been reported previously.

The novel aspect of our data is the potential to compare allelic effects between adolescence and adulthood. Testing for heterogeneity between effect sizes in our adult and adolescent cohorts showed an excess of loci showing p_Het_<0.05 for HDL-C (5 out of 37) and possibly for LDL-C (1 out of 14), but none showed significant heterogeneity (p_Het_<0.05) for triglycerides. The Q-Q plots of observed versus expected distributions of p_Het_ (Figures 1, 2, 3) are consistent with this, but no single locus showed heterogeneity after correction for multiple comparisons (p<0.0014 for HDL-C, p<0.0036 for LDL-C or p<0.0021 for triglycerides). To cover the possibility of differing allelic effects in adolescents and adults at loci which were not discovered in other adult studies or meta-analyses, we checked for significant heterogeneity at all 2.5M genotyped or imputed SNPs, but none was found and nor do the Q-Q plots (Figures S1, S2, S3) show evidence for their existence.

Overall, the largest allelic association effect size (beta) was observed at rs374261 (or rs247616 which is in complete linkage disequilibrium, r^2^ = 1) of *CETP* gene on chromosome 16, in both adolescents and adults. The effect estimates of rs374261 [C] on HDL-C in adolescents and adults were comparable (adolescents: −0.027 mmol/L (equivalent to −1.09 mg/dL) and adults: −0.037 mmol/L (equivalent to −1.49 mg/dL). Even though the estimates obtained in this study have the same directional effect as previous studies, our estimates were much lower. The effect obtained by Teslovich et al. [3] for rs3764261 in adults was −3.39 mg/dL and Smith et al. [8] was −2.99 mg/dL for rs247616 in adolescents. A recent longitudinal genome-wide study on the Bogalusa cohort examined the association between genetic factors and development of CVD risk factors from childhood to adulthood and reported rs247616 on chromsome 16 in *CETP* and rs445925 on chromosome 19 in *APOE* to have significant time-dependent effects (that is, SNP×Age interaction effects) on HDL-C and LDL-C respectively [8]. Our results at *CETP* (p_Het_ = 0.027 for rs374261, r2 = 1.0 with rs247616) confirms the Bogalusa study interaction effect for HDL-C, but we did not find adult-adolescent heterogeneity for LDL-C at *APOE*.

Our other results may also be contrasted with those reported from the longitudinal, repeated-measures Bogalusa study [8]. Our numbers in both the adolescent and the adult groups are substantially greater, but we have insufficient subjects studied at both ages (adolescent and adult) for useful repeated-measures analysis across these stages of life. The Bogalusa study showed SNP×age interactions at p<5×10^−8^ for triglycerides at two SNPs (rs7890572 in *IL1RAPL1* on the X chromosome and rs12280753 between *CADM1* and *BUD13* on chromosome 11) and suggestive interactions (p<10^−6^) for triglycerides at rs13290397 (*PSAT1/CHCHD9*, chromosome 9), rs6726786 (*FSHR/NRXN1*, chromosome 2) and rs12330441 (*IL20RB/SOX14*, chromosome 3). They also found suggestive (again, p<10^−6^) interaction results for LDL-C at rs8073909 (*AKAP1/MSI2*, chromosome 17) and rs11258628 (*FRMD4A*, chromosome 10). As none of these loci were reported as significant in the Global Lipids Consortium meta-analysis and were therefore not prioritised in our comparison, we checked them for heterogeneity between our adults and adolescents but only found rs8073909 on chromosome 17 (*AKAP1/MSI2*) to show significant heterogeneity (p_Het_<0.05) for LDL. We also examined the allelic effects for these SNPs across the seven age groups in our study. We noted that there is an increasing trend of allelic effects in minor allele across age groups for rs8073909 (Figure S4). However, as shown in the Figures, there are no significant differences between allelic effects between the different age groups.

One limitation in our study is our adolescent data did not account for pubertal status. As it is known that during puberty the changes of lipid levels differ between male and female adolescents [26], [27], this is potentially relevant but information on puberty status was not available. However, we did make adjustments for age, sex and age×sex interaction.

There is evidence of lipid changes during therapy for endometriosis patients [28]. To confirm the results in Table 2, we performed a heterogeneity test between samples that consisted of adults from the endometriosis study and others (that is, excluding the endometriosis adults) to ensure that our results are not biased by including participants from that study. We did not find any significant heterogeneity (p_Het_>0.05) between this and the other studies (Table S8). In relation to the other studies (related to alcohol, smoking and anxiety), the focus of these studies was on causes of the condition in the community and the subjects on whom we base our results are similar in relevant respects to the general community. In assessing differences between genetic causes of variation in adolescence and adulthood we suggest that it is better to be inclusive of people with poor lifestyles or pre-clinical disease rather than use only ‘healthy’ controls. Only a small proportion of the participants will be clinically affected cases.

In summary, our study suggests that there is an excess of loci showing nominally significant heterogeneity (p_Het_<0.05) between effect sizes in adults and adolescents for HDL-C. These loci associate differently with lipid traits in adolescents compared to adults. However, no locus showing strong evidence of heterogeneity was found. The Bogalusa study reported seven possible loci showing significant or suggestive SNP×Age interaction effects but our study found only one locus out of their seven showing a significant heterogeneity p-value<0.05. Larger studies with more detailed characterisation of age-related and pubertal changes across childhood and adolescence will be required to reach firm conclusions about age-dependent variation in allelic effects on plasma lipids.

## Supporting Information

Figure S1 **Quantile-quantile plot of observed against expected heterogeneity P-value for allelic associations with HDL-C for all SNP with 95% confidence interval (grey shaded area).**
(TIF)

Figure S2 **Quantile-quantile plot of observed against expected heterogeneity P-value for allelic associations with LDL-C for all SNP with 95% confidence interval (grey shaded area).**
(TIF)

Figure S3 **Quantile-quantile plot of observed against expected heterogeneity P-value for allelic associations with triglycerides for all SNP with 95% confidence interval (grey shaded area).**
(TIF)

Figure S4 Alleleic effects and Error bars SE for each estimated effect of minoar allele by age group at (A) rs247616 (*CETP*) on HDL-C; (B) rs445925 (*APOE*) on LDL-C; (C) rs8073909 (*AKAP1/MSI2*) on LDL-C; (D) rs11258628 (*FRMD4A*) on LDL-C; (E) rs7890572 (IL1RAPL1) on triglycerides; (F) rs12280753 (*CADM1*) on triglycerides; (G) rs13290397 (*PSAT1/CHCHD9*) on triglycerides; (H) rs6726786 (*FSH/NRXN1*) on triglycerides and (I) rs12330441 (*IL20RB/SOX14*) on triglycerides.
(PDF)

Table S1 Association of loci significant with at least one lipid traits in adolescents (n = 2,336), comparing results from the Global lipids Consortium (Teslovich et al) for adult.
(PDF)

Table S2 Heterogeneity P-values within adolescents and adults in HDL-C in 98 SNPs examined.
(PDF)

Table S3 Heterogeneity P-values within adolescents and adults in LDL-C in 98 SNPs examined.
(PDF)

Table S4 Heterogeneity P-values within adolescents and adults in triglycerides in 98 SNPs examined.
(PDF)

Table S5 Heterogeneity test between adolescents and adults in HDL-C in 98 SNPs examined.
(PDF)

Table S6 Heterogeneity test between adolescents and adults in LDL-C in 98 SNPs examined.
(PDF)

Table S7 Heterogeneity test between adolescents and adults in triglycerides in 98 SNPs examined.
(PDF)

Table S8 The locus/phenotype combinations of EN-others heterogeneity tests reported Table 2.
(PDF)
