# Supplementary figures and images for: Evidence of Differential Allelic Effects between Adolescents and Adults for Plasma High-Density Lipoprotein
Source: PLoS One. 2012 Apr 18;7(4):e35605. doi: 10.1371/journal.pone.0035605 (PMC3329456; doi:10.1371/journal.pone.0035605)

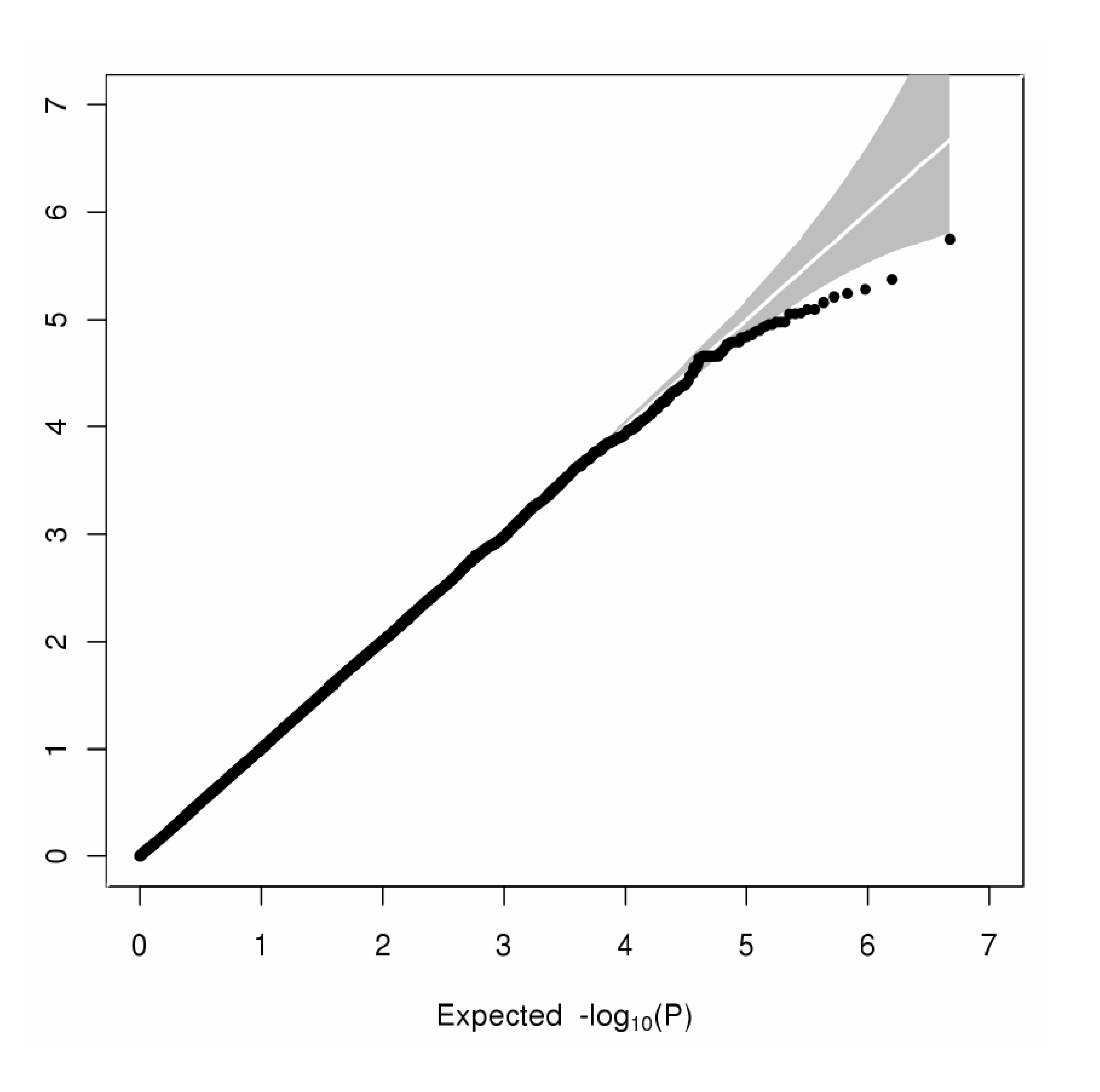

Supplement: Figure S1 — Quantile-quantile plot of observed against expected heterogeneity P-value for allelic associations with HDL-C for all SNP with 95% confidence interval (grey shaded area). (TIF) [file pone.0035605.s001.tif]

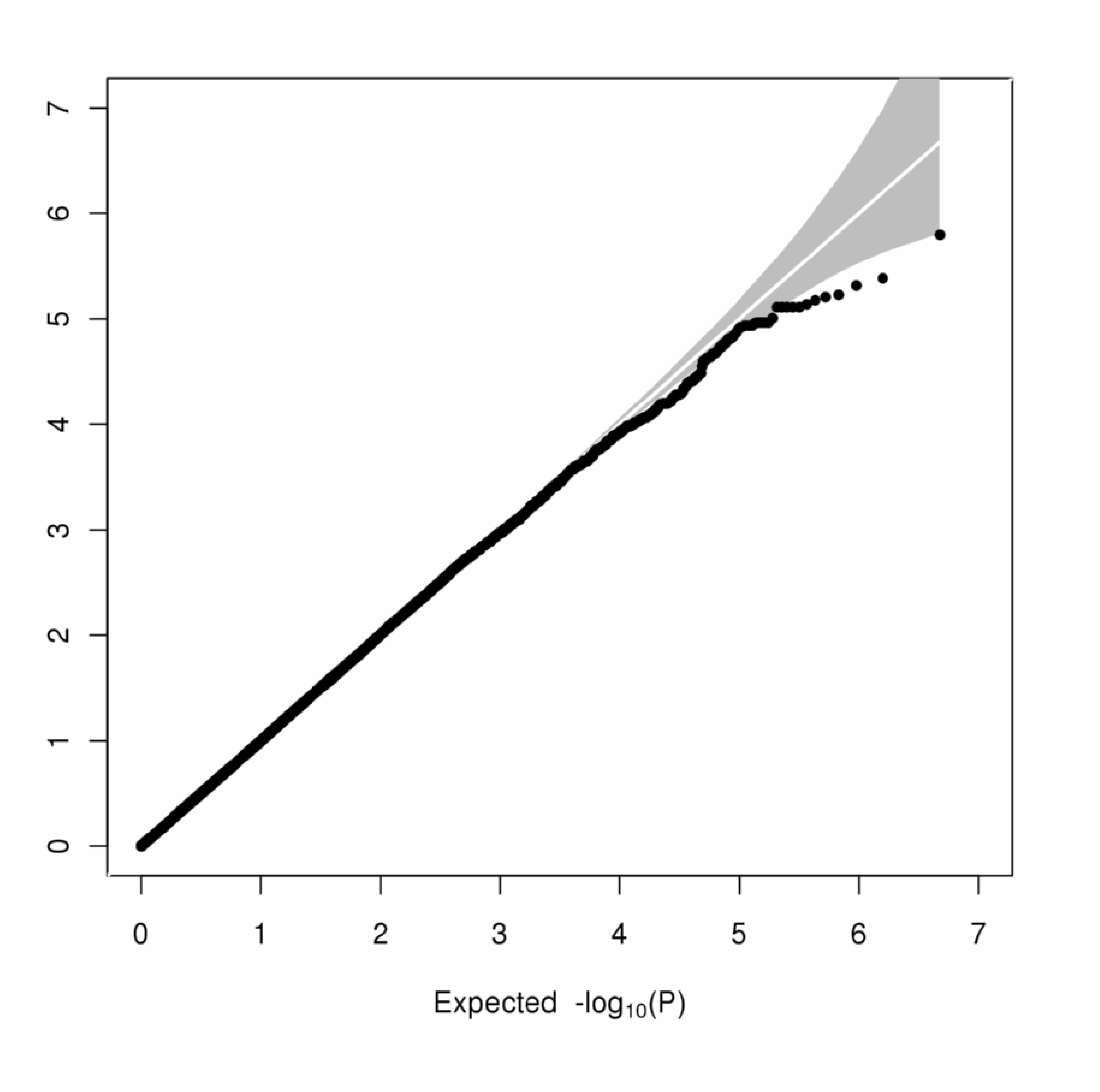

Supplement: Figure S2 — Quantile-quantile plot of observed against expected heterogeneity P-value for allelic associations with LDL-C for all SNP with 95% confidence interval (grey shaded area). (TIF) [file pone.0035605.s002.tif]

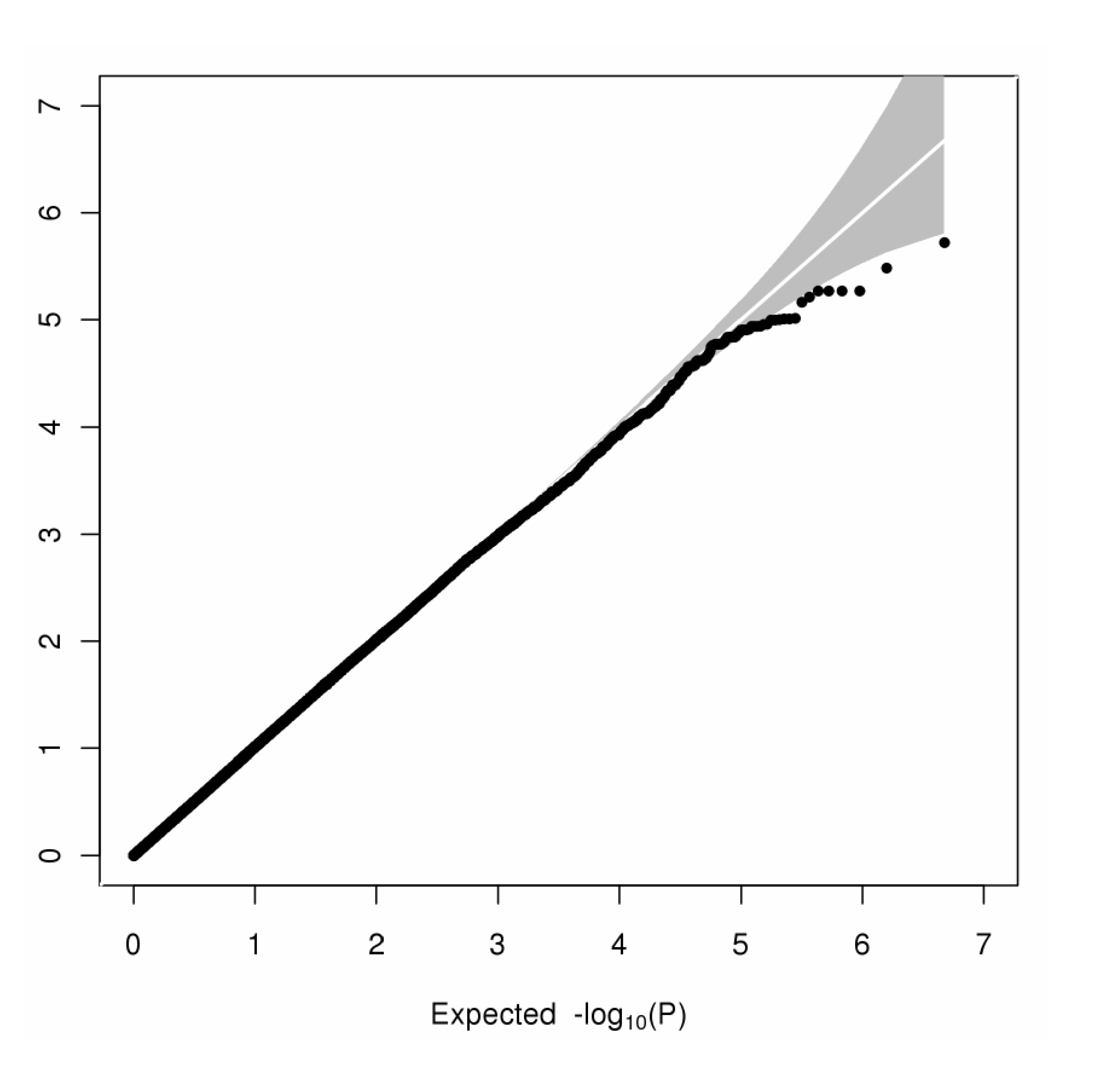

Supplement: Figure S3 — Quantile-quantile plot of observed against expected heterogeneity P-value for allelic associations with triglycerides for all SNP with 95% confidence interval (grey shaded area). (TIF) [file pone.0035605.s003.tif]

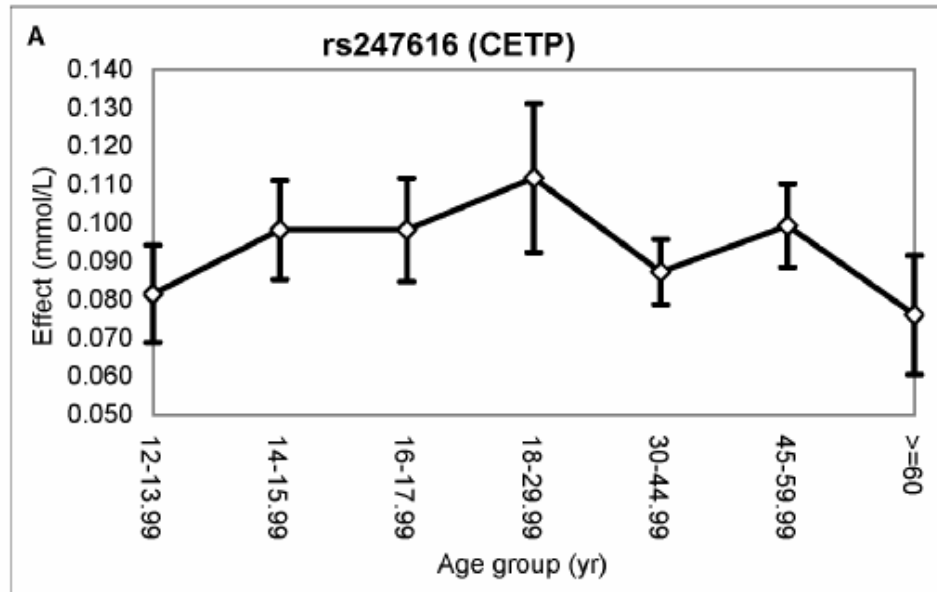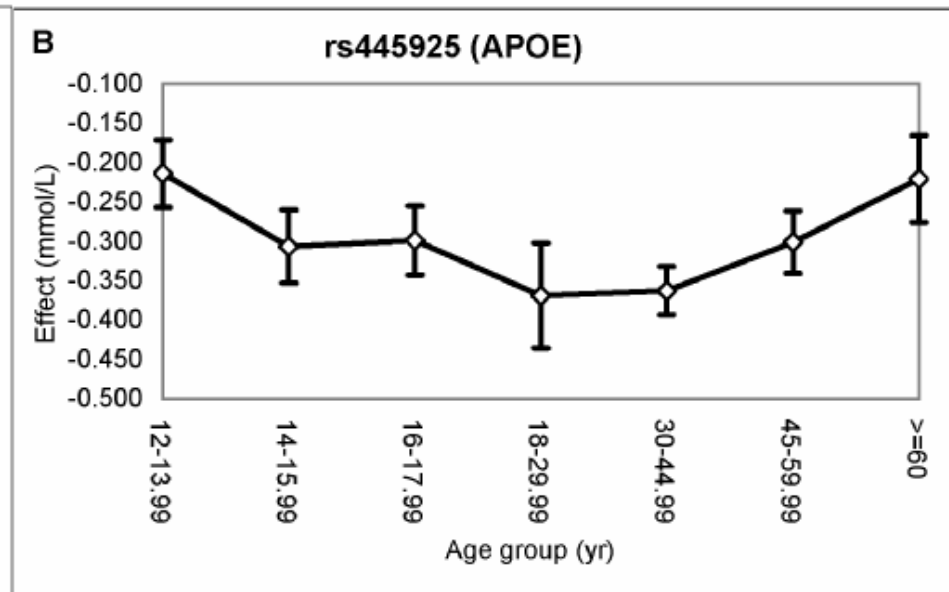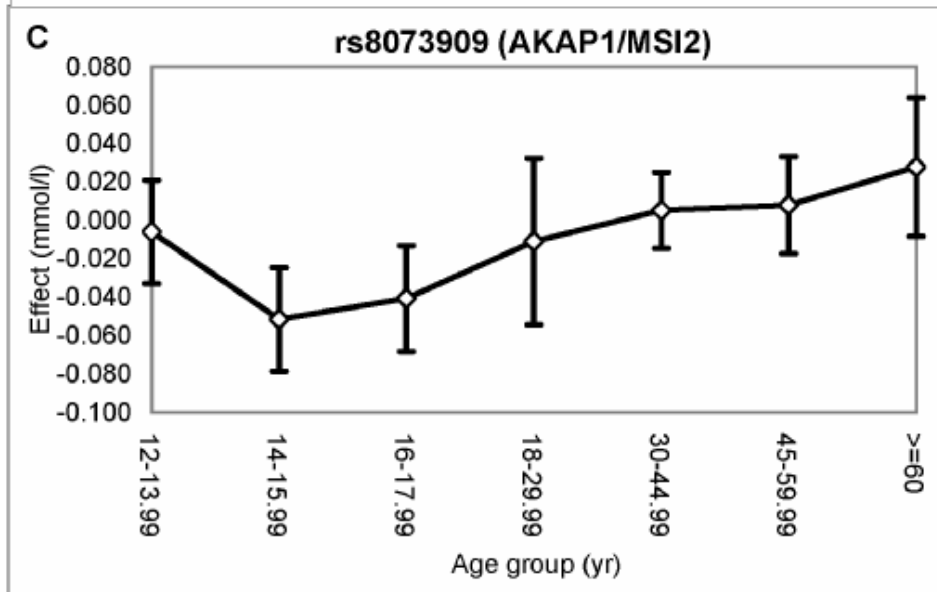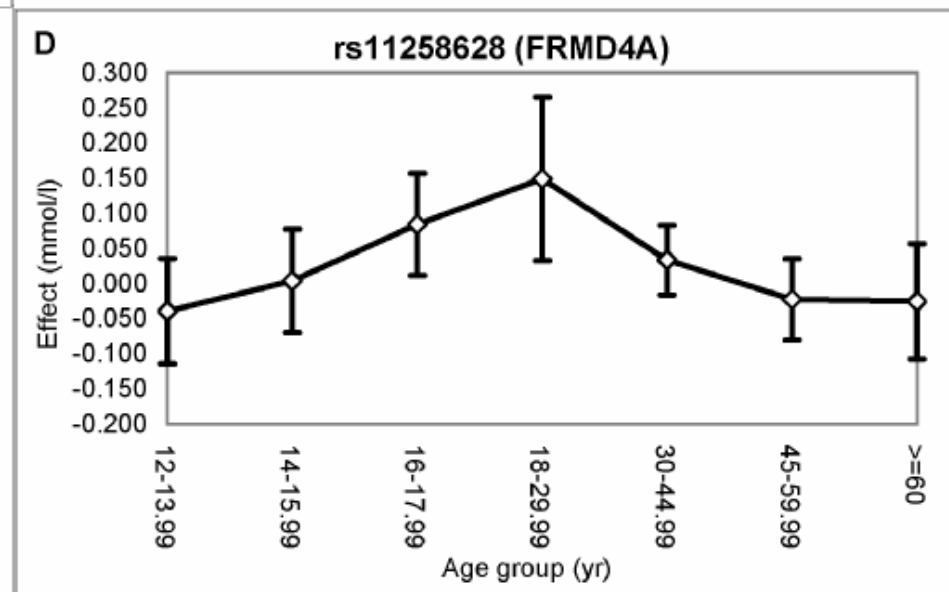

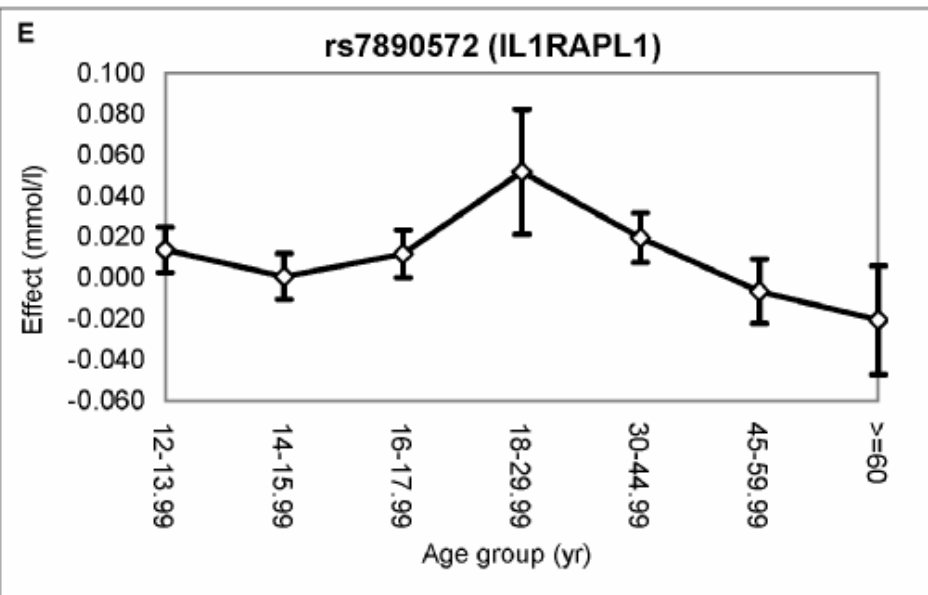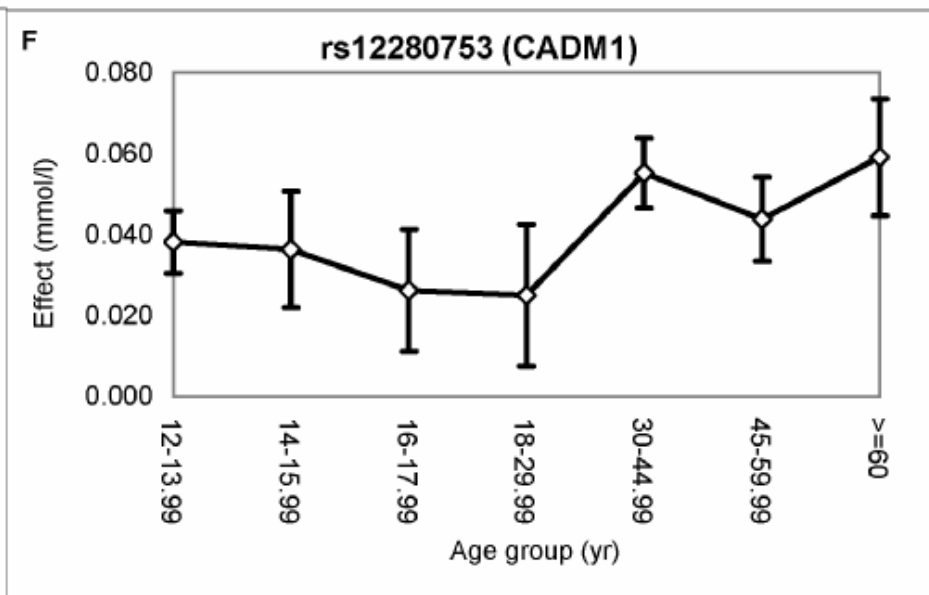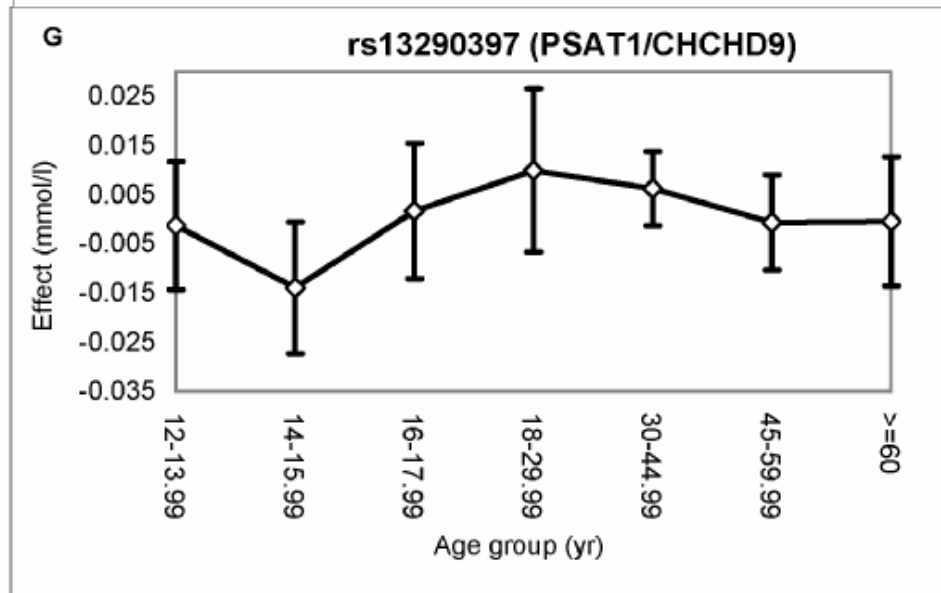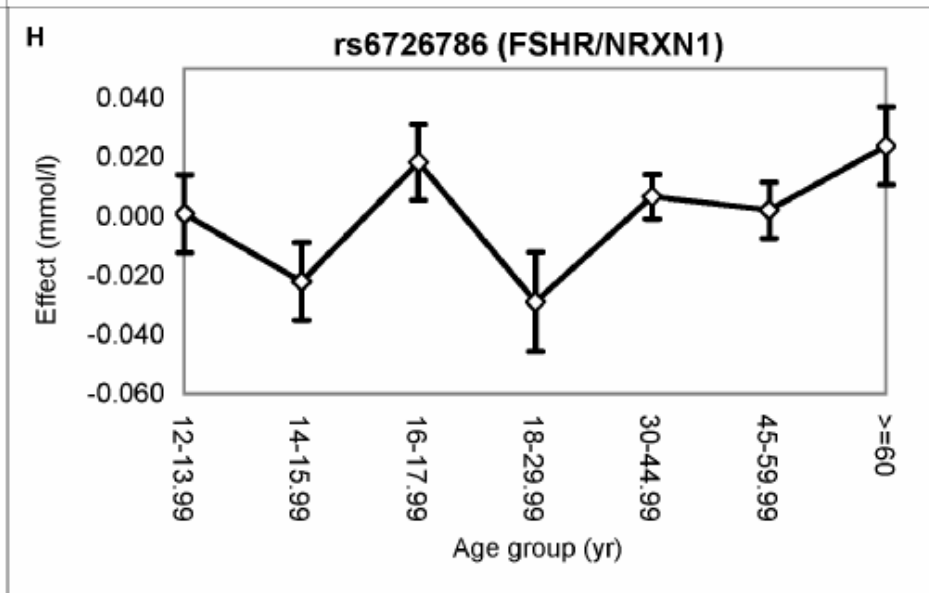

I

# rs12330441 (IL20RB/SOX14)

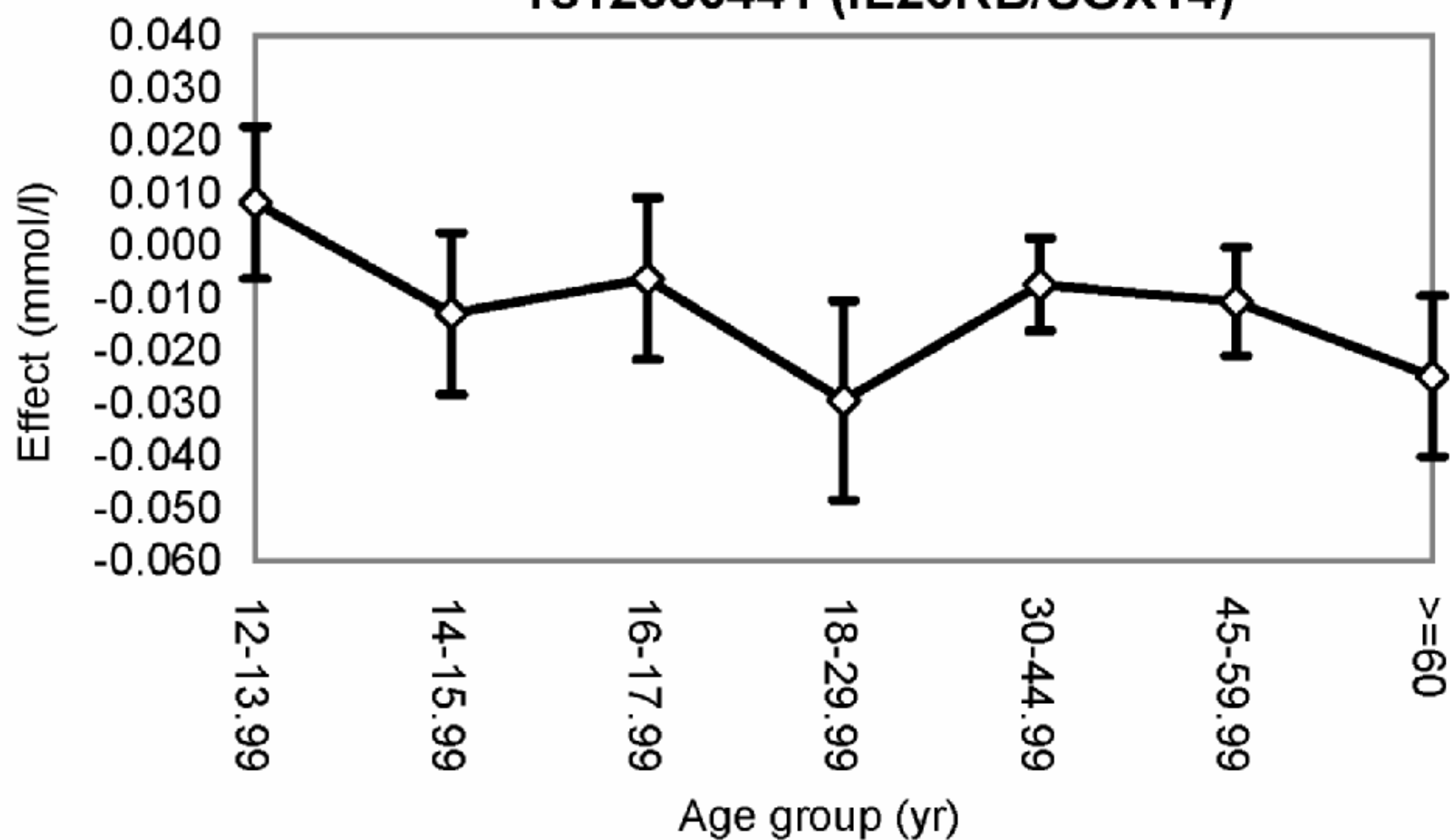

Supplement: Figure S4 — Alleleic effects and Error bars SE for each estimated effect of minoar allele by age group at (A) rs247616 (CETP) on HDL-C; (B) rs445925 (APOE) on LDL-C; (C) rs8073909 (AKAP1/MSI2) on LDL-C; (D) rs11258628 (FRMD4A) on LDL-C; (E) rs7890572 (IL1RAPL1) on triglycerides; (F) rs12280753 (CADM1) on triglycerides; (G) rs13290397 (PSAT1/CHCHD9) on triglycerides; (H) rs6726786 (FSH/NRXN1) on triglycerides and (I) rs12330441 (IL20RB/SOX14) on triglycerides. (PDF) [file pone.0035605.s004.pdf]
