# Supplementary material for: Evidence of Differential Allelic Effects between Adolescents and Adults for Plasma High-Density Lipoprotein
Source: PLoS One. 2012 Apr 18;7(4):e35605. doi: 10.1371/journal.pone.0035605 (PMC3329456; doi:10.1371/journal.pone.0035605)
Supplement: Table S1 — Association of loci significant with at least one lipid traits in adolescents (n = 2,336), comparing results from the Global lipids Consortium (Teslovich et al) for adult. (PDF) [file pone.0035605.s005.pdf]

Table S1. Association of loci significant with at least one lipid traits in adolescents (n=2,336), comparing results from the Global lipids Consortium (Teslovich et al) for adults.

**Global Lipids Consortium Results**								**Our results (adolescents)**								
								**HDL-C**			**LDL-C**			**Triglycerides**		
**Locus**	**Chr**	**SNP**	**Ref Allele**	**Lead trait**	**Betaξ**	**P-Value**	**Other traits**	**Beta**	**SE**	**P-value**	**Beta**	**SE**	**P-value**	**Beta**	**SE**	**P-value**
ANGPTL3	1	rs2131925	T	TG	0.056	9x10^{-43}	LDL	0.012	0.010	0.220	0.042	0.022	0.060	0.013	0.006	**0.033**
LDLRAP1	1	rs12027135	T	TC	0.031	4x10^{-11}	LDL	0.007	0.009	0.490	0.064	0.021	**0.002**	0.004	0.006	0.470
SORT1	1	rs629301	T	LDL	0.141	1x10^{-170}	TC	-0.032	0.011	**0.004**	0.131	0.024	**1.10x 10^{-07}**	0.007	0.007	0.350
ABCG5/8	2	rs4299376	T	LDL	-0.069	2x10^{-47}	TC	0.025	0.010	**0.015**	-0.046	0.023	**0.045**	-0.006	0.006	0.370
APOB	2	rs1042034	T	TG	0.068	1x10^{-45}	HDL	-0.018	0.012	0.137	0.056	0.026	**0.033**	0.011	0.008	0.130
APOB	2	rs1367117	G	LDL	-0.101	4x10^{-114}	TC	-0.006	0.010	0.540	-0.067	0.023	**0.003**	0.005	0.006	0.430
GCKR	2	rs1260326	C	TG	-0.099	6x10^{-133}	TC	-0.004	0.009	0.700	-0.032	0.021	0.140	-0.020	0.006	**0.0009**
KLHL8	4	rs442177	T	TG	0.025	9x10^{-12}		-0.004	0.009	0.670	-0.017	0.021	0.420	0.012	0.006	**0.042**
SLC39A8	4	rs13107325	C	HDL	0.021	7x10^{-11}		0.038	0.017	**0.024**	-0.057	0.037	0.130	-0.011	0.011	0.310
ARL15	5	rs6450176	G	HDL	0.012	5x10^{-08}		0.016	0.011	0.134	-0.056	0.024	**0.022**	-0.016	0.007	0.018
MAP3K1	5	rs9686661	C	TG	-0.029	1x10-^{10}		-0.001	0.011	0.920	-0.063	0.026	**0.015**	-0.012	0.007	0.094
TIMD4	5	rs6882076	C	TC	0.050	7x10^{-28}	LDL, TG	-0.005	0.010	0.580	0.050	0.022	**0.025**	0.016	0.006	**0.009**
C6orf106	6	rs2814982	C	TC	0.047	5x10^{-11}		0.017	0.016	0.290	0.012	0.036	0.740	-0.027	0.010	**0.008**
LPA	6	rs1084651	G	HDL	-0.049	3x10^{-08}		0.027	0.013	**0.034**	0.051	0.028	0.071	0.001	0.008	0.920
MYLIP	6	rs3757354	C	LDL	0.036	1x10^{-11}	TC	-0.005	0.012	0.690	0.053	0.026	**0.041**	0.015	0.007	**0.036**
MLXIPL	7	rs17145738	C	TG	0.105	6x10^{-58}	HDL	-0.028	0.015	0.058	0.029	0.033	0.390	0.027	0.009	**0.004**
TYW1B	7	rs13238203	C	TG	0.089	1x10^{-09}		-0.020	0.029	0.490	0.084	0.064	0.190	0.040	0.018	**0.027**
CYP7A1	8	rs2081687	C	TC	-0.031	2x10^{-12}	LDL	0.012	0.010	0.220	-0.055	0.023	**0.012**	-0.011	0.006	0.074
LPL	8	rs12678919	A	TG	0.154	2x10^{-115}	HDL	-0.045	0.015	**0.003**	0.006	0.034	0.860	0.025	0.010	**0.009**
NAT2	8	rs1495741	A	TG	-0.032	5x10^{-14}	TC	0.015	0.011	0.200	-0.034	0.025	0.180	-0.008	0.007	0.290
TRIB1	8	rs2954029	A	TG	0.064	3x10^{-55}	LDL,HDL	-0.013	0.009	0.170	-0.015	0.021	0.460	0.014	0.006	**0.017**

Global Lipids Consortium Results								Our results (adolescents)								
								**HDL-C**			**LDL-C**			**Triglycerides**		
**Locus**	**Chr**	**SNP**	**Ref Allele**	**Lead trait**	**Betaξ**	**P-Value**	**Other traits**	**Beta**	**SE**	**P-value**	**Beta**	**SE**	**P-value**	**Beta**	**SE**	**P-value**
GPAM	10	rs2255141	G	TC	-0.029	$2x10^{-10}$	LDL	-0.025	0.010	**0.017**	0.029	0.023	0.210	-0.003	0.007	0.710
APOA1	11	rs964184	C	TG	-0.192	$7x10^{-240}$	HDL, LDL	0.023	0.014	0.112	-0.023	0.032	0.490	-0.044	0.009	**$1.20 x 10^{-06}$**
FADS1-2-3	11	rs174546	C	TG	-0.043	$5x10^{-24}$	HDL, LDL	0.006	0.010	0.540	0.080	0.023	**0.00045**	-0.005	0.006	0.460
HNF1A	12	rs1169288	A	TC	-0.036	$1x10^{-14}$	LDL	0.020	0.010	0.046	-0.028	0.022	0.210	-0.016	0.006	0.012
LRP1	12	rs11613352	C	TG	0.031	$4x10^{-10}$	HDL	-0.014	0.011	0.190	0.034	0.024	0.160	0.014	0.007	**0.039**
MVK	12	rs7134594	T	HDL	0.011	$7x10^{-15}$		0.024	0.009	**0.008**	0.017	0.021	0.400	0.003	0.006	0.660
SBNO1	12	rs4759375	C	HDL	-0.022	$7x10^{-09}$		-0.013	0.020	0.500	-0.025	0.044	0.570	-0.025	0.012	**0.046**
FRMD5	15	rs2929282	A	TG	-0.058	$2x10^{-11}$		0.067	0.023	**0.004**	0.009	0.052	0.870	-0.032	0.015	**0.033**
LIPC	15	rs1532085	G	HDL	-0.036	$3x10^{-96}$	TC, TG	-0.032	0.009	**0.001**	-0.048	0.021	**0.027**	-0.008	0.006	0.210
CETP	16	rs3764261	C	HDL	-0.085	$7x10^{-380}$	LDL, TG	-0.095	0.010	**$9.80x 10^{-22}$**	0.006	0.022	0.800	0.008	0.006	0.170
CMIP	16	rs2925979	C	HDL	0.011	$2x10^{-11}$		-0.007	0.011	0.530	0.051	0.024	**0.032**	0.004	0.007	0.530
LIPG	18	rs7241918	T	HDL	0.033	$3x10^{-49}$	TC	0.043	0.012	**0.00029**	0.015	0.027	0.590	0.005	0.008	0.510
MC4R	18	rs12967135	G	HDL	0.011	$7x10^{-09}$		0.039	0.011	**0.001**	-0.001	0.025	0.960	-0.003	0.007	0.660
ANGPTL4	19	rs7255436	A	HDL	0.011	$3x10^{-08}$		0.024	0.009	**0.013**	0.024	0.021	0.240	0.005	0.006	0.400
APOE	19	rs4420638	A	LDL	-0.179	$9x10^{-147}$	TC, HDL	0.027	0.014	**0.048**	-0.141	0.031	**$4.30 x 10^{-06}$**	-0.017	0.009	**0.045**
APOE	19	rs439401	C	TG	0.062	$1x10^{-30}$		-0.009	0.010	0.370	0.026	0.022	0.240	0.021	0.006	**0.0008**
CILP2	19	rs10401969	T	TC	0.119	$3x10^{-38}$	TG, LDL	-0.033	0.018	0.057	0.124	0.039	**0.001**	0.028	0.011	**0.011**
LDLR	19	rs6511720	G	LDL	0.175	$4x10^{-117}$	TC	0.029	0.015	**0.043**	0.021	0.033	0.520	-0.011	0.009	0.230
PLTP	20	rs6065906	T	HDL	0.023	$2x10^{-22}$	TG	0.034	0.012	**0.005**	0.025	0.028	0.360	-0.004	0.008	0.600

ξ Beta for triglycerdes in Teslovich were estimated as percent changes due to a single copy of the minor allele (see Supplementary Table 2 in Teslovich paper) and effect in mg/dL was convert to mmol/L.
