# Supplementary material for: Evidence of Differential Allelic Effects between Adolescents and Adults for Plasma High-Density Lipoprotein
Source: PLoS One. 2012 Apr 18;7(4):e35605. doi: 10.1371/journal.pone.0035605 (PMC3329456; doi:10.1371/journal.pone.0035605)
Supplement: Table S2 — Heterogeneity P-values within adolescents and adults in HDL-C in 98 SNPs examined. (PDF) [file pone.0035605.s006.pdf]

Table S2. Heterogeneity P-values within adolescents and adults in HDL-C in 98 SNPs examined.

				Within Adolescent				Within Adult			
Locus	Chr	SNP	Ref Allele	Beta	SE	Direction	Heterogeneity p-value	Beta	SE	Direction	Heterogeneity p-value
ANGPTL3	1	rs2131925	T	0.037	0.025	++-	0.415	0.023	0.015	++--	0.271
EVI5	1	rs7515577	A	0.072	0.032	+++	0.684	0.029	0.018	++--	0.246
GALNT2	1	rs4846914	A	0.071	0.025	+++	0.948	0.065	0.015	++++	0.144
IRF2BP2	1	rs514230	A	0.029	0.024	+-+	0.342	-0.010	0.014	+--+	0.821
LDLRAP1	1	rs12027135	A	-0.011	0.024	-++	0.422	-0.035	0.014	----	0.288
MOSC1	1	rs2642442	T	-0.013	0.027	-+-	0.296	0.025	0.016	++-+	0.063
PABPC4	1	rs4660293	A	-0.016	0.029	-+-	0.421	0.061	0.018	++++	0.563
PCSK9	1	rs2479409	A	-0.031	0.029	---	0.667	-0.006	0.017	-+-+	0.560
SORT1	1	rs629301	T	-0.079	0.029	---	0.921	-0.046	0.017	----	0.524
ZNF648	1	rs1689800	A	0.003	0.026	++-	0.650	0.010	0.015	+-++	0.630
ABCG5/8	2	rs4299376	T	0.090	0.026	+++	0.633	0.001	0.015	+0--	0.875
APOB	2	rs1042034	T	-0.065	0.031	---	0.422	-0.051	0.017	----	0.864
APOB	2	rs1367117	A	0.012	0.026	-+-	0.623	-0.021	0.015	+--+	0.751
COBLL1	2	rs10195252	T	-0.034	0.024	+--	0.232	-0.018	0.015	---+	0.676
COBLL1	2	rs12328675	T	-0.092	0.035	---	0.549	-0.049	0.022	---+	0.477
GCKR	2	rs1260326	T	-0.005	0.025	--+	0.892	-0.023	0.014	----	0.187
IRS1	2	rs2972146	T	-0.026	0.025	---	0.780	-0.040	0.015	----	0.759
MSL2L1	3	rs645040	T	-0.080	0.029	---	0.928	-0.051	0.017	----	0.950
RAF1	3	rs2290159	C	-0.019	0.028	+--	0.167	-0.005	0.017	++--	0.630
KLHL8	4	rs442177	T	-0.014	0.025	---	0.999	0.013	0.015	+++-	0.466
SLC39A8	4	rs13107325	T	-0.102	0.044	---	0.600	-0.077	0.025	+---	0.120
ARL15	5	rs6450176	A	-0.053	0.029	---	0.593	-0.027	0.017	--++	0.247
HMGCR	5	rs12916	T	-0.007	0.025	--+	0.394	0.004	0.015	+-+-	0.343
MAP3K1	5	rs9686661	T	0.011	0.030	++-	0.895	-0.033	0.018	--+-	0.388
TIMD4	5	rs6882076	T	0.014	0.026	+-+	0.650	-0.020	0.015	----	0.543
C6orf106	6	rs2814944	A	-0.027	0.033	+--	0.352	-0.037	0.020	---+	0.590
C6orf106	6	rs2814982	T	-0.023	0.042	+--	0.474	-0.032	0.025	----	0.934
CITED2	6	rs605066	T	0.019	0.025	+--	0.124	0.041	0.015	+++-	0.342
FRK	6	rs9488822	A	-0.017	0.025	--+	0.345	0.039	0.015	++++	0.503
HFE	6	rs1800562	A	0.064	0.048	+-+	0.024	0.049	0.027	++++	0.674
HLA	6	rs2247056	T	-0.047	0.026	---	0.837	0.007	0.016	--++	0.634
HLA	6	rs3177928	A	0.085	0.034	+++	0.492	0.000	0.020	-+--	0.636

				Within Adolescent				Within Adult			
Locus	Chr	SNP	Ref Allele	Beta	SE	Direction	Heterogeneity p-value	Beta	SE	Direction	Heterogeneity p-value
LPA	6	rs1084651	A	-0.072	0.033	---	0.891	-0.012	0.019	-0--	0.832
LPA	6	rs1564348	T	0.015	0.031	+++	0.977	-0.010	0.019	-++-	0.695
MYLIP	6	rs3757354	T	0.015	0.031	-++	0.297	-0.022	0.018	----	0.949
DNAH11	7	rs12670798	T	-0.018	0.028	+--	0.541	-0.012	0.017	----	0.879
KLF14	7	rs4731702	T	0.019	0.024	+-+	0.703	0.043	0.014	++++	0.076
MLXIPL	7	rs17145738	T	0.084	0.039	+++	0.992	0.090	0.022	++++	0.737
TYW1B	7	rs13238203	T	0.058	0.075	++-	0.776	-0.005	0.040	+--+	0.342
CYP7A1	8	rs2081687	T	-0.039	0.026	---	0.874	0.002	0.015	++--	0.345
LPL	8	rs12678919	A	-0.167	0.039	---	0.202	-0.161	0.024	----	0.086
NAT2	8	rs1495741	A	0.038	0.030	++-	0.280	-0.003	0.017	-+-+	0.553
PINX1	8	rs11776767	C	0.011	0.025	++-	0.762	-0.008	0.015	+++-	0.031
PLEC1	8	rs11136341	A	0.039	0.026	+++	0.969	-0.004	0.015	-+0-	0.558
PPP1R3B	8	rs9987289	A	-0.104	0.044	---	0.393	-0.139	0.025	----	0.431
TRIB1	8	rs2954029	A	-0.041	0.024	---	0.595	-0.033	0.014	----	0.408
TRPS1	8	rs2293889	T	0.025	0.025	-++	0.505	-0.038	0.015	----	0.885
TRPS1	8	rs2737229	A	0.002	0.026	0+-	0.706	-0.006	0.016	+-++	0.229
ABCA1	9	rs1883025	T	-0.017	0.028	-+-	0.448	-0.086	0.016	----	0.962
TTC39B	9	rs581080	C	0.096	0.032	+++	0.476	0.024	0.019	+-++	0.519
CYP26A1	10	rs2068888	A	-0.013	0.024	---	0.998	0.022	0.014	++++	0.966
GPAM	10	rs2255141	A	0.063	0.028	+++	0.443	0.021	0.016	-+++	0.505
JMJD1C	10	rs10761731	A	-0.067	0.024	---	0.757	0.022	0.015	++++	0.511
AMPD3	11	rs2923084	A	-0.064	0.032	---	0.921	0.037	0.019	++++	0.728
APOA1	11	rs964184	C	0.065	0.038	+++	0.538	0.134	0.021	++++	0.113
FADS1-2-3	11	rs174546	T	-0.020	0.026	++-	0.334	-0.062	0.015	----	0.744
LRP4	11	rs3136441	T	0.003	0.037	++-	0.546	-0.016	0.021	-+--	0.379
SPTY2D1	11	rs10128711	T	-0.053	0.028	---	0.968	0.029	0.016	-+++	0.824
ST3GAL4	11	rs11220462	A	0.000	0.036	+-+	0.685	-0.015	0.021	-+--	0.685
UBASH3B	11	rs7941030	T	-0.062	0.025	---	0.457	-0.020	0.015	+-0-	0.524
BRAP	12	rs11065987	A	0.036	0.025	+++	0.938	0.042	0.015	++++	0.593
HNF1A	12	rs1169288	A	0.038	0.026	++-	0.167	-0.018	0.016	--+-	0.535
LRP1	12	rs11613352	T	0.055	0.029	+++	0.439	0.036	0.017	-+++	0.722
MVK	12	rs7134594	T	0.074	0.024	+++	0.793	0.002	0.014	++--	0.561
PDE3A	12	rs7134375	A	0.016	0.024	-++	0.624	0.045	0.014	-+++	0.093
SBNO1	12	rs4759375	T	0.021	0.049	++-	0.945	0.042	0.030	++++	0.908

				Within Adolescent				Within Adult			
Locus	Chr	SNP	Ref Allele	Beta	SE	Direction	Heterogeneity p-value	Beta	SE	Direction	Heterogeneity p-value
SCARB1	12	rs838880	T	-0.037	0.027	---	0.872	-0.034	0.016	---+	0.654
ZNF664	12	rs4765127	T	0.010	0.026	++-	0.073	0.084	0.015	++++	0.434
NYNRIN	14	rs8017377	A	-0.012	0.024	--+	0.315	0.008	0.014	+-+-	0.819
CAPN3	15	rs2412710	A	-0.095	0.089	--+	0.140	-0.049	0.054	--+-	0.215
FRMD5	15	rs2929282	A	0.176	0.061	+++	0.338	0.002	0.036	-+-+	0.348
LACTB	15	rs2652834	A	0.033	0.031	++-	0.636	-0.016	0.018	-+--	0.622
LIPC	15	rs1532085	A	0.108	0.025	+++	0.543	0.093	0.015	++++	0.184
CETP	16	rs3764261	A	0.316	0.026	+++	0.640	0.236	0.015	++++	0.417
CMIP	16	rs2925979	T	0.011	0.027	+++	0.952	-0.035	0.016	----	0.591
CTF1	16	rs11649653	C	-0.045	0.026	---	0.619	-0.009	0.015	--+-	0.802
HPR	16	rs2000999	A	0.000	0.032	-++	0.691	-0.005	0.019	-0-+	0.706
LCAT	16	rs16942887	A	0.022	0.039	+-+	0.903	0.077	0.023	++++	0.966
ABCA8	17	rs4148008	C	0.031	0.026	+++	0.857	0.006	0.016	-++-	0.375
OSBPL7	17	rs7206971	A	0.045	0.024	+++	0.659	0.033	0.014	++++	0.625
PGS1	17	rs4129767	A	0.084	0.024	+++	0.333	-0.008	0.014	+-++	0.637
STARD3	17	rs11869286	C	0.028	0.025	-++	0.616	0.059	0.015	++++	0.064
LIPG	18	rs7241918	T	0.118	0.031	+++	0.789	0.094	0.019	++++	0.794
MC4R	18	rs12967135	A	-0.122	0.029	---	0.822	-0.037	0.017	--+-	0.149
ANGPTL4	19	rs7255436	A	0.055	0.024	+++	0.927	0.034	0.014	-+++	0.177
APOE	19	rs439401	T	0.039	0.025	++-	0.216	0.037	0.015	++++	0.315
APOE	19	rs4420638	A	0.083	0.036	++-	0.058	0.118	0.020	++++	0.914
CILP2	19	rs10401969	T	-0.104	0.046	---	0.862	-0.010	0.027	---+	0.570
FLJ36070	19	rs492602	A	-0.013	0.024	+--	0.838	0.026	0.014	-++0	0.330
LDLR	19	rs6511720	T	-0.116	0.038	---	0.049	0.027	0.023	-+++	0.092
LILRA3	19	rs386000	C	-0.001	0.030	+-+	0.420	0.055	0.017	++++	0.726
LOC55908	19	rs737337	T	0.066	0.046	+++	0.766	0.070	0.027	++++	0.557
ERGIC3	20	rs2277862	T	-0.065	0.034	---	0.438	-0.043	0.021	---+	0.174
MAFB	20	rs2902940	A	-0.013	0.026	+--	0.205	-0.036	0.015	----	0.688
PLTP	20	rs6065906	T	0.119	0.032	+++	0.684	0.060	0.018	+++-	0.422
TOP1	20	rs6029526	A	0.041	0.024	+-+	0.151	0.002	0.014	+--+	0.663
PLA2G6	22	rs5756931	T	0.003	0.025	++-	0.838	-0.024	0.015	----	0.816
UBE2L3	22	rs181362	T	-0.081	0.031	--+	0.093	-0.029	0.019	+-+-	0.020

Numbers in ‘Beta’ and ‘SE’ columns are in standard deviation (SD) unit. The SD unit for adolescents and adults are 0.292 and 0.397.
Adolescents: Age group 1-3 (n=1593, n=1356, n=1350 respectively); Adults: Age group 4-7 (n=1055, n=5198, n=3305, n=1580 respectively)
