# Supplementary material for: Evidence of Differential Allelic Effects between Adolescents and Adults for Plasma High-Density Lipoprotein
Source: PLoS One. 2012 Apr 18;7(4):e35605. doi: 10.1371/journal.pone.0035605 (PMC3329456; doi:10.1371/journal.pone.0035605)
Supplement: Table S3 — Heterogeneity P-values within adolescents and adults in LDL-C in 98 SNPs examined. (PDF) [file pone.0035605.s007.pdf]

Table S3. Heterogeneity P-values within adolescents and adults in LDL-C in 98 SNPs examined.

				Within Adolescent				Within Adult			
Locus	Chr	SNP	Ref Allele	Beta	SE	Direction	Heterogeneity p-value	Beta	SE	Direction	Heterogeneity p-value
ANGPTL3	1	rs2131925	T	0.049	0.026	+++	0.722	0.012	0.015	+++-	0.032
EVI5	1	rs7515577	A	-0.003	0.032	--+	0.609	-0.007	0.018	--+-	0.844
GALNT2	1	rs4846914	A	-0.027	0.025	--+	0.499	-0.024	0.015	-+--	0.058
IRF2BP2	1	rs514230	A	0.006	0.024	++-	0.233	-0.052	0.014	----	0.505
LDLRAP1	1	rs12027135	A	-0.069	0.024	---	0.386	0.003	0.015	-+-+	0.656
MOSC1	1	rs2642442	T	0.039	0.027	++-	0.352	0.011	0.016	--++	0.177
PABPC4	1	rs4660293	A	-0.043	0.029	---	0.612	0.013	0.018	-+++	0.932
PCSK9	1	rs2479409	A	-0.075	0.029	---	0.668	-0.044	0.017	+---	0.393
SORT1	1	rs629301	T	0.178	0.029	+++	0.593	0.150	0.018	++++	0.127
ZNF648	1	rs1689800	A	0.001	0.026	+--	0.144	-0.028	0.015	---+	0.588
ABCG5/8	2	rs4299376	T	-0.032	0.026	---	0.985	-0.050	0.016	----	0.794
APOB	2	rs1367117	A	0.121	0.027	+++	0.532	0.128	0.016	++++	0.421
APOB	2	rs1042034	T	0.083	0.031	+++	0.527	0.039	0.018	+++-	0.185
COBLL1	2	rs10195252	T	-0.059	0.024	---	0.404	0.025	0.015	+++-	0.045
COBLL1	2	rs12328675	T	-0.060	0.035	-+-	0.202	0.004	0.022	++--	0.648
GCKR	2	rs1260326	T	0.044	0.025	+++	0.970	0.010	0.015	+-+-	0.381
IRS1	2	rs2972146	T	-0.002	0.025	--+	0.641	0.015	0.015	-+++	0.771
MSL2L1	3	rs645040	T	0.031	0.029	--+	0.192	0.036	0.017	++++	0.988
RAF1	3	rs2290159	C	-0.019	0.028	-+-	0.446	-0.003	0.017	--++	0.723
KLHL8	4	rs442177	T	-0.025	0.025	---	0.829	-0.010	0.015	---+	0.092
SLC39A8	4	rs13107325	T	0.122	0.045	+++	0.474	-0.020	0.025	---+	0.065
ARL15	5	rs6450176	A	0.080	0.029	+++	0.970	0.024	0.017	+++-	0.486
HMGCR	5	rs12916	T	-0.010	0.025	-+-	0.861	-0.067	0.015	+---	0.012
MAP3K1	5	rs9686661	T	0.085	0.030	+++	0.711	0.038	0.022	++?+	0.877
TIMD4	5	rs6882076	T	-0.060	0.026	---	0.619	-0.045	0.015	+---	0.551
C6orf106	6	rs2814944	A	-0.018	0.033	-+-	0.824	-0.013	0.020	+---	0.857
C6orf106	6	rs2814982	T	0.012	0.042	++-	0.319	-0.052	0.025	----	0.957
CITED2	6	rs605066	T	-0.051	0.025	-+-	0.282	-0.014	0.015	--+-	0.912
FRK	6	rs9488822	A	0.029	0.025	-++	0.606	-0.005	0.018	++?-	0.317
HFE	6	rs1800562	A	-0.036	0.049	---	0.990	-0.068	0.027	---+	0.028
HLA	6	rs3177928	A	0.057	0.035	+++	0.980	0.074	0.020	-+++	0.096
HLA	6	rs2247056	T	-0.015	0.026	-0-	0.883	-0.049	0.016	----	0.519

				Within Adolescent				Within Adult			
Locus	Chr	SNP	Ref Allele	Beta	SE	Direction	Heterogeneity p-value	Beta	SE	Direction	Heterogeneity p-value
LPA	6	rs1084651	A	-0.076	0.033	---	0.930	-0.009	0.020	++-+	0.109
LPA	6	rs1564348	T	-0.012	0.031	--+	0.710	-0.029	0.020	--+-	0.589
MYLIP	6	rs3757354	T	-0.124	0.031	---	0.137	-0.024	0.018	---+	0.770
DNAH11	7	rs12670798	T	0.002	0.028	0-+	0.221	-0.005	0.017	0+--	0.664
KLF14	7	rs4731702	T	-0.023	0.025	---	0.759	-0.036	0.015	----	0.796
MLXIPL	7	rs17145738	T	-0.056	0.039	---	0.505	0.018	0.023	++++	0.994
TYW1B	7	rs13238203	T	-0.123	0.076	---	0.684	0.083	0.041	++++	0.273
CYP7A1	8	rs2081687	T	0.067	0.026	+++	0.409	0.003	0.015	+++-	0.065
LPL	8	rs12678919	A	0.017	0.039	+++	0.992	-0.034	0.024	----	0.823
NAT2	8	rs1495741	A	-0.027	0.030	--+	0.660	0.009	0.018	-+-+	0.378
PINX1	8	rs11776767	C	0.020	0.025	+++	0.907	-0.008	0.015	++--	0.579
PLEC1	8	rs11136341	A	0.001	0.026	-+0	0.968	-0.001	0.015	+-+-	0.673
PPP1R3B	8	rs9987289	A	-0.041	0.044	---	0.973	-0.058	0.030	--?+	0.210
TRIB1	8	rs2954029	A	-0.027	0.025	---	0.858	0.029	0.015	++--	0.115
TRPS1	8	rs2737229	A	0.060	0.026	+++	0.928	0.040	0.016	+++-	0.183
TRPS1	8	rs2293889	T	-0.005	0.025	+--	0.341	0.026	0.015	++++	0.422
ABCA1	9	rs1883025	T	-0.042	0.028	---	0.879	-0.010	0.017	+---	0.728
TTC39B	9	rs581080	C	0.009	0.032	+-+	0.496	0.010	0.019	-++-	0.606
CYP26A1	10	rs2068888	A	0.031	0.024	+++	0.662	-0.001	0.015	-+-+	0.309
GPAM	10	rs2255141	A	-0.053	0.028	---	0.779	0.014	0.016	++++	0.876
JMJD1C	10	rs10761731	A	-0.034	0.024	-+-	0.377	-0.026	0.015	---+	0.295
AMPD3	11	rs2923084	A	0.018	0.032	+-+	0.723	0.019	0.019	--++	0.076
APOA1	11	rs964184	C	-0.022	0.038	--+	0.506	-0.060	0.026	+-?-	0.089
FADS1-2-3	11	rs174546	T	-0.146	0.027	---	0.572	-0.061	0.015	----	0.915
LRP4	11	rs3136441	T	-0.035	0.037	---	0.816	0.052	0.021	++++	0.693
SPTY2D1	11	rs10128711	T	-0.043	0.028	+--	0.213	-0.013	0.017	+---	0.704
ST3GAL4	11	rs11220462	A	0.015	0.036	+-+	0.718	0.030	0.021	++++	0.587
UBASH3B	11	rs7941030	T	0.030	0.025	+-+	0.364	-0.002	0.018	+-?-	0.464
BRAP	12	rs11065987	A	0.037	0.025	-++	0.117	0.028	0.015	++-+	0.192
HNF1A	12	rs1169288	A	-0.065	0.026	---	0.875	-0.072	0.016	----	0.715
LRP1	12	rs11613352	T	-0.052	0.029	---	0.502	-0.015	0.017	+---	0.932
MVK	12	rs7134594	T	0.015	0.024	++-	0.890	0.028	0.014	++++	0.900
PDE3A	12	rs7134375	A	-0.017	0.024	--+	0.285	0.000	0.015	-+-+	0.862
SBNO1	12	rs4759375	T	0.067	0.050	-++	0.286	0.055	0.031	+++-	0.109

				Within Adolescent				Within Adult			
Locus	Chr	SNP	Ref Allele	Beta	SE	Direction	Heterogeneity p-value	Beta	SE	Direction	Heterogeneity p-value
SCARB1	12	rs838880	T	-0.011	0.027	+--	0.837	-0.028	0.019	--?-	0.382
ZNF664	12	rs4765127	T	-0.001	0.026	+--	0.992	-0.013	0.015	---+	0.046
NYNRIN	14	rs8017377	A	-0.021	0.024	---	0.973	0.036	0.017	-+?+	0.220
CAPN3	15	rs2412710	A	-0.014	0.089	+--	0.204	0.051	0.054	+++-	0.712
FRMD5	15	rs2929282	A	0.051	0.061	-++	0.240	-0.021	0.036	---+	0.399
LACTB	15	rs2652834	A	-0.031	0.031	--+	0.302	0.004	0.019	++-+	0.828
LIPC	15	rs1532085	A	0.043	0.025	+++	0.914	0.012	0.015	+++-	0.856
CETP	16	rs3764261	A	-0.009	0.026	+--	0.505	-0.039	0.016	----	0.930
CMIP	16	rs2925979	T	-0.072	0.027	---	0.474	0.007	0.016	-+--	0.600
CTF1	16	rs11649653	C	0.008	0.026	++-	0.960	0.021	0.015	+++-	0.164
HPR	16	rs2000999	A	0.009	0.032	+-+	0.741	0.045	0.019	+++-	0.482
LCAT	16	rs16942887	A	-0.005	0.039	-++	0.369	-0.013	0.023	-+--	0.889
ABCA8	17	rs4148008	C	-0.016	0.027	---	0.767	-0.020	0.016	+---	0.461
OSBPL7	17	rs7206971	A	0.013	0.024	++-	0.863	0.024	0.015	-++-	0.568
PGS1	17	rs4129767	A	0.034	0.024	-++	0.306	0.049	0.015	++++	0.783
STARD3	17	rs11869286	C	0.004	0.025	-+-	0.494	-0.007	0.015	---+	0.899
LIPG	18	rs7241918	T	-0.005	0.031	-+-	0.850	-0.017	0.019	+---	0.935
MC4R	18	rs12967135	A	0.015	0.030	-++	0.782	0.030	0.017	-+++	0.595
ANGPTL4	19	rs7255436	A	0.028	0.025	+++	0.856	0.004	0.015	+--+	0.329
APOE	19	rs4420638	A	-0.231	0.036	---	0.588	-0.154	0.021	---0	0.017
APOE	19	rs439401	T	-0.044	0.025	---	0.583	-0.018	0.015	---+	0.304
CILP2	19	rs10401969	T	0.233	0.046	+++	0.130	0.119	0.028	-+++	0.120
FLJ36070	19	rs492602	A	-0.070	0.024	---	0.676	-0.051	0.014	+---	0.111
LDLR	19	rs6511720	T	-0.062	0.038	---	0.597	-0.176	0.023	----	0.224
LILRA3	19	rs386000	C	-0.010	0.030	-++	0.176	-0.001	0.018	--++	0.573
LOC55908	19	rs737337	T	0.013	0.047	+-+	0.410	0.017	0.028	+-++	0.502
ERGIC3	20	rs2277862	T	0.004	0.034	-++	0.014	-0.038	0.021	----	0.498
MAFB	20	rs2902940	A	0.050	0.027	+++	0.894	0.005	0.016	--++	0.040
PLTP	20	rs6065906	T	0.027	0.032	+++	0.953	0.002	0.019	---+	0.246
TOP1	20	rs6029526	A	0.035	0.024	+-+	0.268	0.017	0.015	++-+	0.175
PLA2G6	22	rs5756931	T	-0.028	0.025	---	0.707	-0.010	0.015	--++	0.560
UBE2L3	22	rs181362	T	0.034	0.031	+++	0.904	-0.024	0.019	+---	0.878

Numbers in ‘Beta’ and ‘SE’ columns are in standard deviation (SD) unit. The SD unit for adolescents and adults are 0.658 and 0.912 respectively.
Adolescents: Age group 1-3 (n=1366, n=1353, n=1349 respectively); Adults: Age group 4-7 (n=1045, n=5050, n=3160, n=1521 respectively)
