# Supplementary material for: Evidence of Differential Allelic Effects between Adolescents and Adults for Plasma High-Density Lipoprotein
Source: PLoS One. 2012 Apr 18;7(4):e35605. doi: 10.1371/journal.pone.0035605 (PMC3329456; doi:10.1371/journal.pone.0035605)
Supplement: Table S4 — Heterogeneity P-values within adolescents and adults in triglycerides in 98 SNPs examined. (PDF) [file pone.0035605.s008.pdf]

Table S4. Heterogeneity P-values within adolescents and adults in triglycerides in 98 SNPs examined

				Within Adolescent				Within Adult			
Locus	Chr	SNP	Ref Allele	Beta	SE	Direction	Heterogeneity p-value	Beta	SE	Direction	Heterogeneity p-value
ANGPTL3	1	rs2131925	T	0.079	0.025	+++	0.891	0.055	0.015	++++	0.849
EVI5	1	rs7515577	A	0.002	0.032	-++	0.943	-0.023	0.017	---+	0.498
GALNT2	1	rs4846914	A	-0.014	0.025	+--	0.670	-0.071	0.014	----	0.021
IRF2BP2	1	rs514230	A	-0.012	0.024	+--	0.496	0.020	0.014	-++-	0.531
LDLRAP1	1	rs12027135	A	-0.011	0.024	-+-	0.336	0.006	0.014	-+-+	0.188
MOSC1	1	rs2642442	T	-0.012	0.027	-+-	0.106	-0.025	0.016	----	0.706
PABPC4	1	rs4660293	A	-0.012	0.029	+--	0.296	-0.027	0.017	----	0.694
PCSK9	1	rs2479409	A	0.032	0.029	-++	0.236	0.007	0.017	+++-	0.916
SORT1	1	rs629301	T	0.080	0.029	+++	0.619	0.018	0.017	+++-	0.555
ZNF648	1	rs1689800	A	0.010	0.026	+--	0.460	-0.012	0.015	+---	0.232
ABCG5/8	2	rs4299376	T	-0.029	0.026	---	0.606	-0.006	0.015	+--+	0.800
APOB	2	rs1367117	A	-0.009	0.026	+--	0.334	0.026	0.015	++++	0.990
APOB	2	rs1042034	T	0.067	0.031	+++	0.340	0.065	0.017	++++	0.747
COBLL1	2	rs12328675	T	0.069	0.035	+++	0.575	0.050	0.022	-++0	0.249
COBLL1	2	rs10195252	T	0.028	0.024	+-+	0.333	0.033	0.015	-+++	0.538
GCKR	2	rs1260326	T	0.079	0.025	+++	0.897	0.083	0.014	++++	0.829
IRS1	2	rs2972146	T	-0.010	0.025	+--	0.602	0.055	0.015	++++	0.313
MSL2L1	3	rs645040	T	0.054	0.029	+++	0.510	0.058	0.017	++++	0.468
RAF1	3	rs2290159	C	0.012	0.028	-++	0.206	-0.007	0.017	--++	0.065
KLHL8	4	rs442177	T	0.063	0.025	+++	0.108	-0.009	0.014	+--+	0.459
SLC39A8	4	rs13107325	T	0.088	0.045	+++	0.704	0.118	0.025	-+++	0.009
ARL15	5	rs6450176	A	0.095	0.029	+++	0.532	-0.001	0.017	+--+	0.410
HMGCR	5	rs12916	T	0.005	0.025	-++	0.840	0.001	0.015	+--+	0.629
MAP3K1	5	rs9686661	T	0.032	0.030	+++	0.888	0.078	0.018	++++	0.356
TIMD4	5	rs6882076	T	-0.047	0.026	---	0.570	-0.037	0.015	---+	0.127
C6orf106	6	rs2814944	A	0.066	0.033	+++	0.610	0.003	0.020	++0-	0.614
C6orf106	6	rs2814982	T	0.135	0.042	+++	0.612	0.006	0.025	-++-	0.677
CITED2	6	rs605066	T	-0.035	0.025	--+	0.518	-0.037	0.015	----	0.913
FRK	6	rs9488822	A	0.012	0.025	++-	0.823	-0.017	0.015	----	0.909
HFE	6	rs1800562	A	0.037	0.048	+++	0.828	-0.030	0.026	+---	0.680

				Within Adolescent				Within Adult			
Locus	Chr	SNP	Ref Allele	Beta	SE	Direction	Heterogeneity p-value	Beta	SE	Direction	Heterogeneity p-value
HLA	6	rs3177928	A	-0.043	0.035	+--	0.435	-0.001	0.020	--++	0.942
HLA	6	rs2247056	T	-0.013	0.026	+--	0.758	-0.051	0.015	+---	0.338
LPA	6	rs1084651	A	-0.015	0.033	--+	0.325	-0.022	0.019	--+-	0.332
LPA	6	rs1564348	T	0.001	0.031	--+	0.093	0.026	0.019	++++	0.781
MYLIP	6	rs3757354	T	-0.104	0.031	---	0.877	0.025	0.018	++-+	0.251
DNAH11	7	rs12670798	T	0.000	0.028	-++	0.368	0.008	0.017	+++-	0.438
KLF14	7	rs4731702	T	-0.013	0.024	-++	0.566	-0.042	0.014	----	0.173
MLXIPL	7	rs17145738	T	-0.164	0.039	---	0.367	-0.118	0.022	----	0.579
TYW1B	7	rs13238203	T	-0.158	0.076	---	0.772	-0.018	0.040	+-+-	0.180
CYP7A1	8	rs2081687	T	0.049	0.026	+++	0.335	0.035	0.015	++++	0.890
LPL	8	rs12678919	A	0.151	0.039	+++	0.557	0.190	0.023	++++	0.096
NAT2	8	rs1495741	A	-0.040	0.030	---	0.880	-0.016	0.017	--+-	0.627
PINX1	8	rs11776767	C	0.014	0.025	+-+	0.639	0.024	0.015	++-+	0.013
PLEC1	8	rs11136341	A	-0.003	0.026	-+-	0.848	0.002	0.015	--++	0.582
PPP1R3B	8	rs9987289	A	0.040	0.044	-++	0.337	0.039	0.025	-+++	0.134
TRIB1	8	rs2954029	A	0.065	0.024	+++	0.500	0.088	0.014	++++	0.425
TRPS1	8	rs2737229	A	0.033	0.026	+-+	0.559	0.029	0.016	++-+	0.333
TRPS1	8	rs2293889	T	-0.044	0.025	-+-	0.337	-0.016	0.014	--++	0.175
ABCA1	9	rs1883025	T	-0.059	0.028	---	0.453	-0.039	0.016	----	0.249
TTC39B	9	rs581080	C	-0.044	0.032	--+	0.361	0.003	0.019	-++-	0.303
CYP26A1	10	rs2068888	A	0.031	0.024	+++	0.750	-0.040	0.014	+---	0.342
GPAM	10	rs2255141	A	0.021	0.028	-++	0.323	-0.024	0.016	+---	0.713
JMJD1C	10	rs10761731	A	0.013	0.024	++-	0.413	0.017	0.015	++-+	0.226
AMPD3	11	rs2923084	A	-0.024	0.032	-+-	0.734	-0.031	0.019	+---	0.795
APOA1	11	rs964184	C	-0.245	0.038	---	0.423	-0.285	0.021	----	0.410
FADS1-2-3	11	rs174546	T	0.034	0.026	-++	0.585	0.068	0.015	++++	0.964
LRP4	11	rs3136441	T	-0.012	0.037	--+	0.633	0.063	0.021	++++	0.370
SPTY2D1	11	rs10128711	T	-0.005	0.028	-+-	0.665	-0.035	0.016	----	0.984
ST3GAL4	11	rs11220462	A	-0.002	0.036	-+-	0.265	0.010	0.021	-+-+	0.552
UBASH3B	11	rs7941030	T	0.052	0.025	+++	0.540	-0.010	0.015	+-+-	0.402
BRAP	12	rs11065987	A	0.010	0.025	++-	0.285	-0.030	0.014	---+	0.465
HNF1A	12	rs1169288	A	-0.066	0.026	---	0.299	-0.006	0.016	+--+	0.802

				Within Adolescent				Within Adult			
Locus	Chr	SNP	Ref Allele	Beta	SE	Direction	Heterogeneity p-value	Beta	SE	Direction	Heterogeneity p-value
LRP1	12	rs11613352	T	-0.085	0.029	---	0.387	-0.056	0.017	----	0.752
MVK	12	rs7134594	T	0.010	0.024	0+-	0.713	0.001	0.014	-+-+	0.452
PDE3A	12	rs7134375	A	0.026	0.024	++-	0.712	-0.024	0.014	++--	0.024
SBNO1	12	rs4759375	T	0.092	0.049	+++	0.904	-0.013	0.030	++--	0.725
SCARB1	12	rs838880	T	-0.019	0.027	+--	0.031	0.016	0.016	+++-	0.207
ZNF664	12	rs4765127	T	-0.045	0.026	---	0.799	-0.066	0.015	----	0.279
NYNRIN	14	rs8017377	A	0.027	0.024	+++	0.717	-0.007	0.014	+---	0.471
CAPN3	15	rs2412710	A	-0.067	0.090	---	0.890	0.047	0.054	++-+	0.385
FRMD5	15	rs2929282	A	-0.053	0.061	---	0.816	-0.032	0.036	--+-	0.165
LACTB	15	rs2652834	A	0.072	0.031	+++	0.746	-0.004	0.018	+--+	0.873
LIPC	15	rs1532085	A	0.060	0.025	+++	0.915	0.062	0.015	++++	0.154
CETP	16	rs3764261	A	-0.030	0.026	--+	0.624	-0.035	0.015	----	0.723
CMIP	16	rs2925979	T	-0.017	0.027	+--	0.884	0.030	0.016	+++-	0.530
CTF1	16	rs11649653	C	0.024	0.026	+-+	0.401	0.018	0.015	++--	0.118
HPR	16	rs2000999	A	-0.029	0.032	-+-	0.738	0.005	0.018	+++-	0.476
LCAT	16	rs16942887	A	0.044	0.039	+-+	0.217	-0.035	0.023	----	0.945
ABCA8	17	rs4148008	C	-0.003	0.027	--+	0.115	0.008	0.016	++-+	0.771
OSBPL7	17	rs7206971	A	-0.038	0.024	-+-	0.384	-0.033	0.014	----	0.979
PGS1	17	rs4129767	A	0.004	0.024	-+-	0.090	0.006	0.014	-+++	0.702
STARD3	17	rs11869286	C	-0.030	0.025	---	0.813	-0.012	0.015	+---	0.527
LIPG	18	rs7241918	T	0.016	0.031	-++	0.835	-0.028	0.018	----	0.951
MC4R	18	rs12967135	A	0.008	0.029	-++	0.546	0.042	0.017	-++-	0.462
ANGPTL4	19	rs7255436	A	0.005	0.024	+-+	0.812	-0.048	0.014	+---	0.152
APOE	19	rs4420638	A	-0.068	0.036	---	0.451	-0.098	0.020	----	0.578
APOE	19	rs439401	T	-0.095	0.025	---	0.943	-0.073	0.015	----	0.651
CILP2	19	rs10401969	T	0.156	0.046	+++	0.853	0.051	0.027	++++	0.856
FLJ36070	19	rs492602	A	-0.010	0.024	--+	0.695	-0.029	0.014	+---	0.015
LDLR	19	rs6511720	T	0.071	0.038	+++	0.343	0.014	0.023	++-+	0.559
LILRA3	19	rs386000	C	0.019	0.030	+++	0.981	-0.030	0.017	--+-	0.304
LOC55908	19	rs737337	T	0.003	0.046	-++	0.733	-0.012	0.027	+-+-	0.518
ERGIC3	20	rs2277862	T	0.046	0.034	++-	0.253	0.008	0.021	++-+	0.728
MAFB	20	rs2902940	A	-0.011	0.026	---	0.983	0.021	0.015	-+++	0.175

				Within Adolescent				Within Adult			
Locus	Chr	SNP	Ref Allele	Beta	SE	Direction	Heterogeneity p-value	Beta	SE	Direction	Heterogeneity p-value
PLTP	20	rs6065906	T	-0.071	0.032	---	0.968	-0.060	0.018	----	0.762
TOP1	20	rs6029526	A	-0.006	0.024	-+-	0.693	0.012	0.014	-+++	0.887
PLA2G6	22	rs5756931	T	0.039	0.025	+++	0.682	0.000	0.015	+-++	0.620
UBE2L3	22	rs181362	T	0.070	0.031	++-	0.105	-0.023	0.018	-+--	0.168

Numbers in ‘Beta’ and ‘SE’ columns are in standard deviation (SD) unit. To convert to mmol/L, The SD unit for adolescents and adults are 0.183 and 0.216 respectively.
Adolescents: Age group 1-3 (n=1372, n=1359, n=1351 respectively); Adults: Age group 4-7 (n=1060, n=5230, n=3315, n=1586 respectively)
