# Supplementary material for: Evidence of Differential Allelic Effects between Adolescents and Adults for Plasma High-Density Lipoprotein
Source: PLoS One. 2012 Apr 18;7(4):e35605. doi: 10.1371/journal.pone.0035605 (PMC3329456; doi:10.1371/journal.pone.0035605)
Supplement: Table S5 — Heterogeneity test between adolescents and adults in HDL-C in 98 SNPs examined. (PDF) [file pone.0035605.s009.pdf]

Table S5. Heterogeneity test between adolescents and adults in HDL-C in 98 SNPs examined.

				Within Adolescent			Within Adult			Adolescent + Adult				
Locus	Chr	SNP	Ref Allele	Beta	SE	P-value	Beta	SE	P-value	Beta	SE	P-value	Direction	Heterogeneity p-value
ANGPTL3	1	rs2131925	T	0.042	0.034	0.220	0.024	0.016	0.142	0.027	0.015	0.05967	++	0.632
EVI5	1	rs7515577	A	0.070	0.042	0.098	0.026	0.019	0.170	0.034	0.017	0.053	++	0.340
GALNT2	1	rs4846914	A	0.040	0.033	0.240	0.065	0.016	3.30×10^{-05}	0.060	0.014	2.86×10^{-05}	++	0.495
IRF2BP2	1	rs514230	T	-0.056	0.033	0.088	0.014	0.015	0.370	0.002	0.014	0.883	-+	0.053
LDLRAP1	1	rs12027135	T	0.023	0.033	0.490	0.037	0.016	0.016	0.034	0.014	0.01708	++	0.703
MOSC1	1	rs2642442	T	-0.015	0.036	0.680	0.026	0.017	0.119	0.019	0.015	0.228	-+	0.303
PABPC4	1	rs4660293	A	-0.029	0.038	0.450	0.061	0.019	0.001	0.043	0.017	0.011	-+	0.034
PCSK9	1	rs2479409	A	-0.057	0.038	0.137	0.001	0.018	0.960	-0.010	0.016	0.554	-+	0.168
SORT1	1	rs629301	T	-0.111	0.038	0.004	-0.046	0.019	0.014	-0.059	0.017	0.001	--	0.126
ZNF648	1	rs1689800	A	-0.006	0.034	0.860	0.008	0.016	0.630	0.006	0.015	0.706	-+	0.710
ABCG5/8	2	rs4299376	T	0.086	0.035	0.015	-0.002	0.016	0.920	0.013	0.015	0.364	+-	0.022
APOB	2	rs1042034	T	-0.061	0.041	0.137	-0.054	0.019	0.005	-0.055	0.017	0.001354	--	0.877
APOB	2	rs1367117	G	-0.021	0.035	0.540	0.021	0.017	0.210	0.013	0.015	0.396	-+	0.280
COBLL1	2	rs10195252	T	-0.021	0.032	0.510	-0.014	0.016	0.360	-0.015	0.014	0.2819	--	0.845
COBLL1	2	rs12328675	T	-0.076	0.047	0.105	-0.053	0.024	0.025	-0.058	0.021	0.00689	--	0.663
GCKR	2	rs1260326	C	-0.013	0.033	0.700	0.020	0.016	0.210	0.014	0.014	0.341	-+	0.368
IRS1	2	rs2972146	T	-0.046	0.034	0.180	-0.028	0.016	0.080	-0.031	0.015	0.03081	--	0.632
MSL2L1	3	rs645040	T	-0.073	0.039	0.059	-0.046	0.018	0.011	-0.051	0.016	0.002	--	0.530
RAF1	3	rs2290159	G	0.016	0.038	0.670	0.000	0.018	0.990	0.003	0.016	0.857	0	0.704
KLHL8	4	rs442177	T	-0.014	0.033	0.670	0.009	0.016	0.560	0.005	0.014	0.748	-+	0.531
SLC39A8	4	rs13107325	C	0.132	0.058	0.024	0.063	0.026	0.017	0.075	0.024	0.002	++	0.278
ARL15	5	rs6450176	G	0.057	0.038	0.134	0.035	0.018	0.050	0.039	0.016	0.016	++	0.601
HMGCR	5	rs12916	T	0.025	0.033	0.450	0.005	0.016	0.760	0.009	0.014	0.541	++	0.586
MAP3K1	5	rs9686661	C	-0.004	0.040	0.920	0.022	0.019	0.240	0.017	0.017	0.316	-+	0.557
TIMD4	5	rs6882076	C	-0.019	0.034	0.580	0.019	0.016	0.230	0.012	0.015	0.403	-+	0.312
C6orf106	6	rs2814944	G	0.045	0.044	0.310	0.036	0.021	0.093	0.038	0.019	0.04685	++	0.854
C6orf106	6	rs2814982	C	0.059	0.056	0.290	0.021	0.026	0.420	0.028	0.024	0.240	++	0.538
CITED2	6	rs605066	T	0.031	0.033	0.350	0.033	0.016	0.033	0.033	0.014	0.02347	++	0.957
FRK	6	rs9488822	A	-0.029	0.034	0.390	0.034	0.016	0.031	0.023	0.015	0.119	-+	0.094
HFE	6	rs1800562	G	-0.058	0.063	0.360	-0.028	0.029	0.330	-0.033	0.026	0.2069	--	0.665
HLA	6	rs2247056	C	0.038	0.034	0.270	0.000	0.017	0.990	0.008	0.015	0.617	0	0.318
HLA	6	rs3177928	G	-0.080	0.046	0.083	-0.017	0.021	0.420	-0.028	0.019	0.145	--	0.213
LPA	6	rs1084651	G	0.093	0.044	0.034	0.011	0.021	0.600	0.026	0.019	0.167	++	0.093

				Within Adolescent			Within Adult			Adolescent + Adult				
Locus	Chr	SNP	Ref Allele	Beta	SE	P-value	Beta	SE	P-value	Beta	SE	P-value	Direction	Heterogeneity p-value
LPA	6	rs1564348	T	0.005	0.042	0.900	-0.002	0.021	0.930	-0.001	0.019	0.9745	+-	0.882
MYLIP	6	rs3757354	C	-0.016	0.041	0.690	0.021	0.019	0.270	0.015	0.017	0.402	-+	0.413
DNAH11	7	rs12670798	T	-0.030	0.038	0.430	-0.010	0.018	0.570	-0.014	0.016	0.4009	--	0.634
KLF14	7	rs4731702	C	-0.032	0.033	0.320	-0.046	0.015	0.003	-0.044	0.014	0.001408	--	0.699
MLXIPL	7	rs17145738	C	-0.097	0.051	0.058	-0.102	0.024	2.00 x 10^{-05}	-0.101	0.022	3.24 x 10^{-06}	--	0.929
TYW1B	7	rs13238203	C	-0.069	0.100	0.490	-0.012	0.043	0.780	-0.021	0.040	0.597	--	0.601
CYP7A1	8	rs2081687	C	0.042	0.035	0.220	-0.002	0.016	0.890	0.006	0.015	0.700	+-	0.253
LPL	8	rs12678919	A	-0.158	0.053	0.003	-0.162	0.025	1.60 x 10^{-}10	-0.161	0.023	9.86 x 10^{-13}	--	0.946
NAT2	8	rs1495741	A	0.051	0.039	0.200	0.008	0.019	0.680	0.016	0.017	0.342	++	0.322
PINX1	8	rs11776767	G	-0.013	0.033	0.700	0.002	0.016	0.900	-0.001	0.014	0.9526	-+	0.683
PLEC1	8	rs11136341	A	0.042	0.034	0.220	0.006	0.016	0.710	0.013	0.015	0.387	++	0.338
PPP1R3B	8	rs9987289	G	0.097	0.059	0.101	0.139	0.027	2.10 x 10^{-07}	0.132	0.025	8.08 x 10^{-08}	++	0.517
TRIB1	8	rs2954029	A	-0.045	0.033	0.170	-0.033	0.015	0.033	-0.035	0.014	0.01026	--	0.741
TRPS1	8	rs2293889	G	-0.015	0.033	0.660	0.049	0.015	0.002	0.038	0.014	0.005	-+	0.077
TRPS1	8	rs2737229	A	-0.019	0.035	0.590	-0.007	0.017	0.660	-0.009	0.015	0.5435	--	0.758
ABCA1	9	rs1883025	C	0.052	0.037	0.160	0.087	0.018	7.40 x 10^{-07}	0.080	0.016	7.01 x 10^{-07}	++	0.395
TTC39B	9	rs581080	C	0.069	0.043	0.109	0.023	0.020	0.260	0.031	0.018	0.086	++	0.332
CYP26A1	10	rs2068888	G	0.016	0.032	0.630	-0.019	0.015	0.210	-0.013	0.014	0.350	+-	0.322
GPAM	10	rs2255141	G	-0.087	0.036	0.017	-0.028	0.017	0.102	-0.039	0.015	0.012	--	0.138
JMJD1C	10	rs10761731	A	-0.056	0.033	0.085	0.019	0.016	0.220	0.005	0.014	0.743	-+	0.041
AMPD3	11	rs2923084	A	-0.050	0.043	0.250	0.033	0.020	0.104	0.018	0.018	0.315	-+	0.080
APOA1	11	rs964184	C	0.080	0.050	0.112	0.119	0.023	2.30 x 10^{-07}	0.112	0.021	7.91 x 10^{-08}	++	0.479
FADS1-2-3	11	rs174546	C	0.022	0.036	0.540	0.062	0.016	1.30 x 10^{-04}	0.055	0.015	0.000	++	0.310
LRP4	11	rs3136441	T	0.000	0.049	0.990	-0.027	0.022	0.220	-0.023	0.020	0.263	0	0.615
SPTY2D1	11	rs10128711	C	0.040	0.037	0.280	-0.028	0.017	0.115	-0.016	0.015	0.296	+-	0.095
ST3GAL4	11	rs11220462	G	-0.044	0.048	0.360	0.001	0.023	0.970	-0.007	0.021	0.721	-+	0.398
UBASH3B	11	rs7941030	T	-0.058	0.033	0.080	-0.026	0.016	0.098	-0.032	0.014	0.026	--	0.383
BRAP	12	rs11065987	A	0.033	0.033	0.320	0.042	0.016	0.007	0.040	0.014	0.005137	++	0.806
HNF1A	12	rs1169288	A	0.069	0.034	0.046	-0.018	0.017	0.290	-0.001	0.015	0.969	+-	0.022
LRP1	12	rs11613352	C	-0.050	0.038	0.190	-0.025	0.018	0.160	-0.030	0.016	0.069	--	0.552
MVK	12	rs7134594	T	0.085	0.032	0.008	0.010	0.015	0.510	0.024	0.014	0.083	++	0.034
PDE3A	12	rs7134375	C	-0.018	0.033	0.590	-0.041	0.016	0.008	-0.037	0.014	0.011	--	0.531
SBNO1	12	rs4759375	C	-0.047	0.069	0.500	-0.051	0.032	0.113	-0.050	0.029	0.0832	--	0.958
SCARB1	12	rs838880	T	-0.064	0.036	0.074	-0.035	0.017	0.041	-0.040	0.015	0.009	--	0.466

				Within Adolescent			Within Adult			Adolescent + Adult				
Locus	Chr	SNP	Ref Allele	Beta	SE	P-value	Beta	SE	P-value	Beta	SE	P-value	Direction	Heterogeneity p-value
ZNF664	12	rs4765127	G	0.009	0.034	0.800	-0.080	0.016	8.00×10^{-07}	-0.064	0.015	1.03×10^{-05}	+-	0.018
NYNRIN	14	rs8017377	G	-0.001	0.033	0.980	-0.013	0.015	0.410	-0.011	0.014	0.4228	--	0.741
CAPN3	15	rs2412710	G	0.149	0.121	0.220	0.063	0.058	0.280	0.079	0.052	0.131	++	0.522
FRMD5	15	rs2929282	A	0.233	0.081	0.004	0.020	0.039	0.610	0.060	0.035	0.087	++	0.018
LACTB	15	rs2652834	G	-0.032	0.042	0.450	0.008	0.020	0.680	0.001	0.018	0.973	-+	0.390
LIPC	15	rs1532085	G	-0.111	0.033	0.001	-0.093	0.016	3.10×10^{-09}	-0.096	0.014	2.12×10^{-11}	--	0.624
CETP	16	rs3764261	C	-0.330	0.035	9.80×10^{-22}	-0.245	0.016	1.20×10^{-50}	-0.260	0.015	3.09×10^{-71}	--	0.027
CMIP	16	rs2925979	C	-0.023	0.037	0.530	0.035	0.017	0.037	0.025	0.015	0.107	-+	0.154
CTF1	16	rs11649653	C	-0.059	0.034	0.084	-0.011	0.016	0.500	-0.020	0.015	0.174	--	0.202
HPR	16	rs2000999	G	-0.001	0.042	0.980	-0.003	0.020	0.880	-0.003	0.018	0.8842	--	0.966
LCAT	16	rs16942887	G	-0.031	0.052	0.550	-0.085	0.024	0.00042	-0.076	0.022	0.001	--	0.346
ABCA8	17	rs4148008	C	0.023	0.036	0.520	0.008	0.017	0.630	0.011	0.015	0.485	++	0.706
OSBPL7	17	rs7206971	G	-0.041	0.032	0.200	-0.025	0.015	0.102	-0.028	0.014	0.04008	--	0.651
PGS1	17	rs4129767	G	-0.045	0.032	0.160	0.010	0.015	0.500	0.000	0.014	0.995	-+	0.120
STARD3	17	rs11869286	C	0.025	0.034	0.460	0.063	0.016	9.70×10^{-05}	0.056	0.015	0.000	++	0.312
LIPG	18	rs7241918	T	0.150	0.041	0.000	0.082	0.020	4.00×10^{-05}	0.095	0.018	1.23×10^{-07}	++	0.136
MC4R	18	rs12967135	G	0.136	0.039	0.001	0.032	0.018	0.077	0.050	0.016	0.002	++	0.015
ANGPTL4	19	rs7255436	A	0.082	0.033	0.013	0.039	0.015	0.012	0.046	0.014	0.001	++	0.236
APOE	19	rs439401	C	-0.030	0.034	0.370	-0.035	0.016	0.029	-0.034	0.015	0.01852	--	0.894
APOE	19	rs4420638	A	0.094	0.048	0.048	0.117	0.022	5.60×10^{-08}	0.113	0.020	1.60×10^{-08}	++	0.663
CILP2	19	rs10401969	T	-0.116	0.061	0.057	-0.006	0.029	0.820	-0.026	0.026	0.316	--	0.103
FLJ36070	19	rs492602	G	-0.003	0.033	0.930	-0.023	0.015	0.137	-0.020	0.014	0.152	--	0.581
LDLR	19	rs6511720	G	0.102	0.051	0.043	-0.019	0.024	0.440	0.003	0.022	0.892	+-	0.032
LILRA3	19	rs386000	G	-0.017	0.039	0.670	-0.063	0.019	0.001	-0.054	0.017	0.002	--	0.289
LOC55908	19	rs737337	T	0.086	0.062	0.170	0.060	0.029	0.041	0.065	0.026	0.01382	++	0.704
ERGIC3	20	rs2277862	C	0.039	0.045	0.380	0.039	0.022	0.079	0.039	0.020	0.04847	++	1.000
MAFB	20	rs2902940	A	0.013	0.035	0.710	-0.042	0.016	0.010	-0.033	0.015	0.026	+-	0.153
PLTP	20	rs6065906	T	0.120	0.043	0.005	0.067	0.020	0.001	0.076	0.018	2.50×10^{-05}	++	0.264
TOP1	20	rs6029526	T	-0.048	0.032	0.137	-0.005	0.015	0.740	-0.013	0.014	0.348	--	0.224
PLA2G6	22	rs5756931	T	0.006	0.033	0.850	-0.022	0.016	0.170	-0.017	0.014	0.247	+-	0.445
UBE2L3	22	rs181362	C	0.079	0.042	0.061	0.020	0.020	0.320	0.031	0.018	0.087	++	0.205

Numbers in ‘Beta’ and ‘SE’ columns are in standard deviation (SD) unit. The SD unit for adolescents and adults are 0.292 and 0.397 respectively
