# Supplementary material for: Evidence of Differential Allelic Effects between Adolescents and Adults for Plasma High-Density Lipoprotein
Source: PLoS One. 2012 Apr 18;7(4):e35605. doi: 10.1371/journal.pone.0035605 (PMC3329456; doi:10.1371/journal.pone.0035605)
Supplement: Table S6 — Heterogeneity test between adolescents and adults in LDL-C in 98 SNPs examined. (PDF) [file pone.0035605.s010.pdf]

Table S6. Heterogeneity test between adolescents and adults in LDL-C in 98 SNPs examined

				**Within Adolescent**			**Within Adult**			**Adolescent + Adult**				
Locus	Chr	SNP	Ref Allele	Beta	SE	P-value	Beta	SE	P-value	Beta	SE	P-value	Direction	Heterogeneity p-value
ANGPTL3	1	rs2131925	T	0.065	0.034	0.060	0.005	0.016	0.760	0.016	0.015	0.273	++	0.110
EVI5	1	rs7515577	A	0.035	0.042	0.410	-0.008	0.019	0.680	-0.001	0.017	0.968	+-	0.351
GALNT2	1	rs4846914	A	-0.034	0.033	0.310	-0.011	0.016	0.480	-0.015	0.014	0.286	--	0.531
IRF2BP2	1	rs514230	T	-0.002	0.033	0.950	0.044	0.015	0.004	0.036	0.014	0.008	-+	0.204
LDLRAP1	1	rs12027135	T	0.099	0.033	0.002	-0.005	0.016	0.760	0.015	0.014	0.304	-+	0.005
MOSC1	1	rs2642442	T	0.040	0.036	0.260	0.005	0.017	0.780	0.011	0.015	0.459	++	0.379
PABPC4	1	rs4660293	A	-0.027	0.038	0.490	0.016	0.019	0.410	0.007	0.017	0.663	-+	0.312
PCSK9	1	rs2479409	A	-0.069	0.038	0.071	-0.045	0.018	0.012	-0.049	0.016	0.002	--	0.568
SORT1	1	rs629301	T	0.203	0.038	0.000	0.151	0.019	1.90×10^{-15}	0.161	0.017	2.15×10^{-21}	++	0.221
ZNF648	1	rs1689800	A	0.018	0.034	0.590	-0.025	0.016	0.124	-0.017	0.015	0.235	+-	0.253
ABCG5/8	2	rs4299376	T	-0.071	0.035	0.045	-0.051	0.017	0.002	-0.055	0.015	0.000	--	0.607
APOB	2	rs1042034	T	0.087	0.041	0.033	0.027	0.019	0.160	0.038	0.017	0.029	++	0.184
APOB	2	rs1367117	G	-0.104	0.035	0.003	-0.124	0.017	1.90×10^{-13}	-0.120	0.015	3.86×10^{-15}	--	0.607
COBLL1	2	rs10195252	T	-0.055	0.032	0.090	0.026	0.016	0.106	0.010	0.014	0.494	-+	0.024
COBLL1	2	rs12328675	T	-0.030	0.047	0.530	-0.005	0.024	0.820	-0.010	0.021	0.634	--	0.636
GCKR	2	rs1260326	C	-0.049	0.033	0.140	-0.010	0.016	0.520	-0.017	0.014	0.226	--	0.288
IRS1	2	rs2972146	T	0.000	0.034	1.000	0.012	0.016	0.450	0.010	0.015	0.497	0+	0.750
MSL2L1	3	rs645040	T	0.047	0.039	0.220	0.036	0.018	0.050	0.038	0.016	0.020	++	0.798
RAF1	3	rs2290159	G	0.050	0.038	0.190	0.008	0.018	0.670	0.016	0.016	0.335	++	0.318
KLHL8	4	rs442177	T	-0.027	0.033	0.420	-0.004	0.016	0.820	-0.008	0.014	0.561	--	0.531
SLC39A8	4	rs13107325	C	-0.088	0.058	0.130	0.022	0.027	0.420	0.002	0.025	0.922	-+	0.086
ARL15	5	rs6450176	G	-0.087	0.038	0.022	-0.019	0.018	0.300	-0.032	0.016	0.053	--	0.106
HMGCR	5	rs12916	T	-0.030	0.033	0.360	-0.066	0.016	4.70×10^{-05}	-0.059	0.014	3.99×10^{-05}	--	0.326
MAP3K1	5	rs9686661	C	-0.098	0.040	0.015	-0.022	0.019	0.260	-0.036	0.017	0.036	--	0.086
TIMD4	5	rs6882076	C	0.077	0.034	0.025	0.049	0.016	0.003	0.054	0.015	0.000	++	0.456
C6orf106	6	rs2814944	G	0.058	0.044	0.190	0.015	0.022	0.480	0.024	0.020	0.230	++	0.382
C6orf106	6	rs2814982	C	0.018	0.056	0.740	0.058	0.027	0.029	0.051	0.024	0.038	++	0.520
CITED2	6	rs605066	T	-0.039	0.033	0.240	-0.008	0.016	0.600	-0.014	0.014	0.334	--	0.398
FRK	6	rs9488822	A	0.006	0.034	0.860	-0.012	0.016	0.450	-0.009	0.015	0.546	+-	0.632
HFE	6	rs1800562	G	-0.014	0.063	0.830	0.068	0.029	0.019	0.054	0.026	0.042	-+	0.237
HLA	6	rs2247056	C	0.009	0.034	0.790	0.044	0.017	0.010	0.037	0.015	0.015	++	0.357
HLA	6	rs3177928	G	-0.054	0.046	0.240	-0.078	0.021	0.000	-0.074	0.019	0.000	--	0.635
LPA	6	rs1084651	G	0.079	0.044	0.071	0.006	0.021	0.770	0.020	0.019	0.302	++	0.134

				Within Adolescent			Within Adult			Adolescent + Adult				
Locus	Chr	SNP	Ref Allele	Beta	SE	P-value	Beta	SE	P-value	Beta	SE	P-value	Direction	Heterogeneity p-value
LPA	6	rs1564348	T	-0.021	0.042	0.630	-0.032	0.021	0.130	-0.030	0.019	0.113	--	0.815
MYLIP	6	rs3757354	C	0.083	0.041	0.041	0.028	0.020	0.160	0.039	0.018	0.032	++	0.228
DNAH11	7	rs12670798	T	-0.040	0.038	0.300	0.000	0.018	0.990	-0.007	0.016	0.652	0	0.342
KLF14	7	rs4731702	C	0.032	0.033	0.320	0.039	0.016	0.013	0.038	0.014	0.009	++	0.849
MLXIPL	7	rs17145738	C	0.045	0.051	0.390	-0.018	0.024	0.460	-0.007	0.022	0.762	-+	0.264
TYW1B	7	rs13238203	C	0.131	0.100	0.190	-0.072	0.044	0.098	-0.039	0.040	0.332	-+	0.063
CYP7A1	8	rs2081687	C	-0.086	0.035	0.012	0.002	0.016	0.900	-0.013	0.015	0.364	-+	0.022
LPL	8	rs12678919	A	0.009	0.053	0.860	-0.028	0.025	0.270	-0.021	0.023	0.347	+-	0.528
NAT2	8	rs1495741	A	-0.053	0.039	0.180	0.015	0.019	0.420	0.002	0.017	0.909	-+	0.117
PINX1	8	rs11776767	G	-0.006	0.033	0.860	0.013	0.016	0.420	0.009	0.014	0.515	-+	0.604
PLEC1	8	rs11136341	A	0.000	0.034	1.000	-0.010	0.016	0.520	-0.008	0.015	0.572	0	0.790
PPP1R3B	8	rs9987289	G	0.065	0.059	0.270	0.041	0.027	0.132	0.045	0.025	0.066	++	0.712
TRIB1	8	rs2954029	A	-0.024	0.033	0.460	0.031	0.016	0.045	0.021	0.014	0.154	-+	0.134
TRPS1	8	rs2293889	G	-0.002	0.033	0.960	-0.028	0.016	0.076	-0.023	0.014	0.109	--	0.478
TRPS1	8	rs2737229	A	0.043	0.035	0.220	0.043	0.017	0.013	0.043	0.015	0.005	++	1.000
ABCA1	9	rs1883025	C	0.049	0.037	0.180	0.002	0.018	0.890	0.011	0.016	0.497	++	0.253
TTC39B	9	rs581080	C	-0.017	0.043	0.690	0.015	0.020	0.450	0.009	0.018	0.608	-+	0.500
CYP26A1	10	rs2068888	G	-0.029	0.032	0.370	-0.005	0.016	0.760	-0.010	0.014	0.494	--	0.502
GPAM	10	rs2255141	G	0.045	0.036	0.210	-0.015	0.018	0.410	-0.003	0.016	0.852	-+	0.136
JMJD1C	10	rs10761731	A	-0.032	0.033	0.320	-0.025	0.016	0.110	-0.026	0.014	0.067	--	0.849
AMPD3	11	rs2923084	A	0.039	0.043	0.370	0.018	0.020	0.380	0.022	0.018	0.231	++	0.658
APOA1	11	rs964184	C	-0.035	0.050	0.490	-0.073	0.023	0.002	-0.066	0.021	0.001	--	0.490
FADS1-2-3	11	rs174546	C	0.125	0.036	0.000	0.060	0.016	0.000	0.071	0.015	1.32×10^{-06}	++	0.099
LRP4	11	rs3136441	T	0.015	0.049	0.760	0.058	0.023	0.010	0.050	0.021	0.016	++	0.427
SPTY2D1	11	rs10128711	C	0.056	0.037	0.130	0.018	0.018	0.320	0.025	0.016	0.118	++	0.356
ST3GAL4	11	rs11220462	G	-0.018	0.048	0.700	-0.037	0.023	0.110	-0.034	0.021	0.107	--	0.721
UBASH3B	11	rs7941030	T	0.041	0.033	0.220	-0.004	0.016	0.820	0.005	0.014	0.751	+-	0.220
BRAP	12	rs11065987	A	0.032	0.033	0.340	0.026	0.016	0.098	0.027	0.014	0.059	++	0.870
HNF1A	12	rs1169288	A	-0.044	0.034	0.210	-0.078	0.017	3.90×10^{-06}	-0.071	0.015	2.83×10^{-06}	--	0.371
LRP1	12	rs11613352	C	0.053	0.038	0.160	0.012	0.018	0.520	0.020	0.016	0.230	++	0.330
MVK	12	rs7134594	T	0.027	0.032	0.400	0.032	0.016	0.038	0.031	0.014	0.030	++	0.889
PDE3A	12	rs7134375	C	0.000	0.033	0.990	0.002	0.016	0.890	0.002	0.014	0.910	0	0.957
SBNO1	12	rs4759375	C	-0.039	0.069	0.570	-0.035	0.033	0.280	-0.036	0.030	0.230	--	0.958
SCARB1	12	rs838880	T	-0.008	0.036	0.830	-0.021	0.017	0.220	-0.019	0.015	0.226	--	0.744

				Within Adolescent			Within Adult			Adolescent + Adult				
Locus	Chr	SNP	Ref Allele	Beta	SE	P-value	Beta	SE	P-value	Beta	SE	P-value	Direction	Heterogeneity p-value
ZNF664	12	rs4765127	G	0.003	0.034	0.940	0.010	0.016	0.530	0.009	0.015	0.547	++	0.852
NYNRIN	14	rs8017377	G	0.006	0.033	0.860	-0.043	0.016	0.006	-0.034	0.014	0.019	-+	0.182
CAPN3	15	rs2412710	G	-0.093	0.121	0.450	-0.053	0.059	0.370	-0.061	0.053	0.253	--	0.766
FRMD5	15	rs2929282	A	0.014	0.081	0.870	-0.034	0.039	0.390	-0.025	0.035	0.477	+-	0.593
LACTB	15	rs2652834	G	0.014	0.042	0.740	-0.011	0.020	0.590	-0.006	0.018	0.724	-+	0.591
LIPC	15	rs1532085	G	-0.074	0.033	0.027	-0.008	0.016	0.590	-0.021	0.014	0.153	--	0.072
CETP	16	rs3764261	C	0.009	0.034	0.800	0.053	0.017	0.002	0.044	0.015	0.004	++	0.247
CMIP	16	rs2925979	C	0.079	0.037	0.032	-0.016	0.017	0.360	0.001	0.015	0.971	-+	0.020
CTF1	16	rs11649653	C	0.015	0.034	0.670	0.032	0.016	0.052	0.029	0.015	0.046	++	0.651
HPR	16	rs2000999	G	0.000	0.042	1.000	-0.046	0.020	0.021	-0.038	0.018	0.038	0	0.323
LCAT	16	rs16942887	G	0.006	0.052	0.910	0.003	0.024	0.910	0.004	0.022	0.871	++	0.958
ABCA8	17	rs4148008	C	0.016	0.036	0.650	-0.022	0.017	0.200	-0.015	0.015	0.327	+-	0.340
OSBPL7	17	rs7206971	G	-0.019	0.032	0.550	-0.030	0.015	0.056	-0.028	0.014	0.039	--	0.756
PGS1	17	rs4129767	G	-0.024	0.032	0.460	-0.044	0.016	0.005	-0.040	0.014	0.005	--	0.576
STARD3	17	rs11869286	C	-0.010	0.034	0.760	-0.019	0.016	0.260	-0.017	0.015	0.230	--	0.811
LIPG	18	rs7241918	T	0.023	0.042	0.590	-0.027	0.020	0.190	-0.018	0.018	0.325	+-	0.282
MC4R	18	rs12967135	G	-0.002	0.039	0.960	-0.032	0.018	0.080	-0.027	0.016	0.102	--	0.485
ANGPTL4	19	rs7255436	A	0.038	0.033	0.240	0.007	0.016	0.650	0.013	0.014	0.370	++	0.398
APOE	19	rs439401	C	0.040	0.034	0.240	0.023	0.016	0.160	0.026	0.015	0.072	++	0.651
APOE	19	rs4420638	A	-0.219	0.048	0.000	-0.144	0.022	5.70×10^{-11}	-0.157	0.020	4.12×10^{-15}	--	0.156
CILP2	19	rs10401969	T	0.193	0.061	0.001	0.121	0.030	4.30×10^{-05}	0.135	0.027	5.29×10^{-07}	++	0.290
FLJ36070	19	rs492602	G	0.057	0.033	0.081	0.054	0.016	0.001	0.055	0.014	0.000	++	0.935
LDLR	19	rs6511720	G	0.033	0.051	0.520	0.174	0.025	2.70×10^{-12}	0.147	0.022	6.39×10^{-11}	++	0.013
LILRA3	19	rs386000	G	-0.001	0.039	0.990	0.004	0.019	0.810	0.003	0.017	0.859	-+	0.908
LOC55908	19	rs737337	T	0.043	0.063	0.490	0.022	0.030	0.460	0.026	0.027	0.339	++	0.763
ERGIC3	20	rs2277862	C	-0.047	0.045	0.300	0.033	0.023	0.137	0.016	0.021	0.422	-+	0.113
MAFB	20	rs2902940	A	0.060	0.035	0.084	0.010	0.017	0.570	0.020	0.015	0.201	++	0.199
PLTP	20	rs6065906	T	0.039	0.043	0.360	-0.005	0.020	0.810	0.003	0.018	0.876	+-	0.354
TOP1	20	rs6029526	T	-0.059	0.032	0.067	-0.014	0.016	0.360	-0.023	0.014	0.108	--	0.209
PLA2G6	22	rs5756931	T	-0.035	0.033	0.290	-0.006	0.016	0.720	-0.012	0.014	0.424	--	0.429
UBE2L3	22	rs181362	C	-0.061	0.042	0.145	0.024	0.020	0.230	0.008	0.018	0.646	-+	0.068

Numbers in ‘Beta’ and ‘SE’ columns are in standard deviation (SD) unit. The SD unit for adolescents and adults are 0.658 and 0.912 respectively.
