# Supplementary material for: Evidence of Differential Allelic Effects between Adolescents and Adults for Plasma High-Density Lipoprotein
Source: PLoS One. 2012 Apr 18;7(4):e35605. doi: 10.1371/journal.pone.0035605 (PMC3329456; doi:10.1371/journal.pone.0035605)
Supplement: Table S7 — Heterogeneity test between adolescents and adults in triglycerides in 98 SNPs examined. (PDF) [file pone.0035605.s011.pdf]

Table S7. Heterogeneity test between adolescents and adults in triglycerides in 98 SNPs examined.

				**Within Adolescent**			**Within Adult**			**Adolescent + Adult**				
Locus	Chr	SNP	Ref Allele	Beta	SE	P-value	Beta	SE	P-value	Beta	SE	P-value	Direction	Heterogeneity p-value
ANGPTL3	1	rs2131925	T	0.072	0.034	0.033	0.056	0.016	0.001	0.059	0.015	4.73×10^{-05}	++	0.670
EVI5	1	rs7515577	A	0.003	0.042	0.940	-0.026	0.019	0.170	-0.021	0.017	0.224	+-	0.529
GALNT2	1	rs4846914	A	-0.031	0.033	0.350	-0.070	0.016	7.20×10^{-06}	-0.063	0.014	1.38×10^{-05}	--	0.288
IRF2BP2	1	rs514230	T	0.034	0.032	0.290	-0.023	0.015	0.128	-0.013	0.014	0.349	+-	0.107
LDLRAP1	1	rs12027135	T	0.023	0.032	0.470	-0.008	0.015	0.600	-0.002	0.014	0.859	+-	0.380
MOSC1	1	rs2642442	T	-0.017	0.035	0.630	-0.025	0.017	0.134	-0.024	0.015	0.125	--	0.837
PABPC4	1	rs4660293	A	-0.035	0.038	0.360	-0.026	0.019	0.160	-0.028	0.017	0.102	--	0.832
PCSK9	1	rs2479409	A	0.069	0.038	0.068	-0.003	0.018	0.860	0.010	0.016	0.531	+-	0.087
SORT1	1	rs629301	T	0.036	0.038	0.350	0.019	0.019	0.310	0.022	0.017	0.188	++	0.689
ZNF648	1	rs1689800	A	0.029	0.034	0.390	-0.008	0.016	0.610	-0.001	0.015	0.929	+-	0.325
ABCG5/8	2	rs4299376	T	-0.031	0.035	0.370	-0.005	0.016	0.760	-0.010	0.015	0.514	--	0.499
APOB	2	rs1042034	T	0.061	0.041	0.130	0.064	0.019	0.001	0.064	0.017	0.000	++	0.947
APOB	2	rs1367117	G	0.027	0.035	0.430	-0.020	0.017	0.230	-0.011	0.015	0.471	+-	0.227
COBLL1	2	rs10195252	T	0.010	0.032	0.750	0.032	0.016	0.040	0.028	0.014	0.054	++	0.539
COBLL1	2	rs12328675	T	0.085	0.046	0.064	0.052	0.023	0.025	0.059	0.021	0.004	++	0.521
GCKR	2	rs1260326	C	-0.108	0.033	0.0009	-0.079	0.016	3.70×10^{-07}	-0.085	0.014	4.34×10^{-09}	--	0.429
IRS1	2	rs2972146	T	-0.003	0.034	0.930	0.043	0.016	0.007	0.035	0.015	0.017	-+	0.221
MSL2L1	3	rs645040	T	0.033	0.038	0.390	0.053	0.018	0.004	0.049	0.016	0.002	++	0.634
RAF1	3	rs2290159	G	-0.014	0.038	0.710	0.005	0.018	0.770	0.002	0.016	0.926	-+	0.651
KLHL8	4	rs442177	T	0.066	0.033	0.042	-0.006	0.016	0.700	0.008	0.014	0.593	+-	0.050
SLC39A8	4	rs13107325	C	-0.059	0.058	0.310	-0.111	0.026	2.60×10^{-05}	-0.102	0.024	1.62×10^{-05}	--	0.413
ARL15	5	rs6450176	G	-0.089	0.038	0.018	-0.004	0.018	0.810	-0.020	0.016	0.229	--	0.043
HMGCR	5	rs12916	T	-0.001	0.033	0.990	-0.004	0.016	0.790	-0.003	0.014	0.812	--	0.935
MAP3K1	5	rs9686661	C	-0.067	0.040	0.094	-0.075	0.019	9.30×10^{-05}	-0.074	0.017	1.83×10^{-05}	--	0.857
TIMD4	5	rs6882076	C	0.089	0.034	0.009	0.036	0.016	0.024	0.046	0.015	0.002	++	0.158
C6orf106	6	rs2814944	G	-0.054	0.044	0.220	0.005	0.021	0.820	-0.006	0.019	0.754	-+	0.226
C6orf106	6	rs2814982	C	-0.147	0.055	0.008	-0.001	0.026	0.980	-0.028	0.024	0.239	--	0.016
CITED2	6	rs605066	T	-0.030	0.033	0.370	-0.036	0.016	0.022	-0.035	0.014	0.015	--	0.870
FRK	6	rs9488822	A	-0.023	0.033	0.500	-0.014	0.016	0.390	-0.016	0.014	0.275	--	0.806
HFE	6	rs1800562	G	-0.018	0.062	0.770	0.015	0.029	0.610	0.009	0.026	0.730	-+	0.630
HLA	6	rs2247056	C	0.005	0.034	0.890	0.044	0.017	0.009	0.036	0.015	0.017	++	0.305
HLA	6	rs3177928	G	0.029	0.045	0.520	0.020	0.021	0.340	0.022	0.019	0.256	++	0.856
LPA	6	rs1084651	G	0.005	0.043	0.920	0.029	0.021	0.160	0.024	0.019	0.196	++	0.616

				Within Adolescent			Within Adult			Adolescent + Adult				
Locus	Chr	SNP	Ref Allele	Beta	SE	P-value	Beta	SE	P-value	Beta	SE	P-value	Direction	Heterogeneity p-value
LPA	6	rs1564348	T	-0.018	0.042	0.660	0.013	0.021	0.540	0.007	0.019	0.717	-+	0.509
MYLIP	6	rs3757354	C	0.084	0.040	0.036	-0.029	0.019	0.131	-0.008	0.017	0.633	+-	0.011
DNAH11	7	rs12670798	T	0.005	0.038	0.890	0.003	0.018	0.860	0.003	0.016	0.836	++	0.962
KLF14	7	rs4731702	C	0.005	0.032	0.870	0.043	0.015	0.005	0.036	0.014	0.008	++	0.282
MLXIPL	7	rs17145738	C	0.145	0.051	0.004	0.121	0.024	4.00×10^{-07}	0.125	0.022	7.82×10^{-09}	++	0.670
TYW1B	7	rs13238203	C	0.219	0.099	0.027	0.029	0.043	0.490	0.059	0.039	0.134	++	0.078
CYP7A1	8	rs2081687	C	-0.061	0.034	0.074	-0.029	0.016	0.072	-0.035	0.015	0.016	--	0.394
LPL	8	rs12678919	A	0.137	0.052	0.009	0.190	0.025	3.70×10^{-14}	0.180	0.023	1.34×10^{-15}	++	0.358
NAT2	8	rs1495741	A	-0.042	0.039	0.290	-0.029	0.018	0.115	-0.031	0.016	0.056	--	0.762
PINX1	8	rs11776767	G	-0.001	0.033	0.970	-0.023	0.016	0.150	-0.019	0.014	0.191	--	0.549
PLEC1	8	rs11136341	A	-0.007	0.034	0.840	-0.001	0.016	0.930	-0.002	0.015	0.885	--	0.873
PPP1R3B	8	rs9987289	G	-0.090	0.058	0.123	-0.043	0.027	0.106	-0.051	0.025	0.036	--	0.463
TRIB1	8	rs2954029	A	0.077	0.032	0.017	0.085	0.015	2.50×10^{-08}	0.084	0.014	7.64×10^{-10}	++	0.821
TRPS1	8	rs2293889	G	0.036	0.033	0.260	0.009	0.015	0.570	0.014	0.014	0.319	++	0.456
TRPS1	8	rs2737229	A	0.013	0.034	0.700	0.038	0.017	0.026	0.033	0.015	0.030	++	0.511
ABCA1	9	rs1883025	C	0.055	0.036	0.130	0.030	0.018	0.088	0.035	0.016	0.030	++	0.535
TTC39B	9	rs581080	C	-0.035	0.043	0.410	0.013	0.020	0.530	0.005	0.018	0.806	-+	0.312
CYP26A1	10	rs2068888	G	-0.014	0.031	0.660	0.034	0.015	0.029	0.025	0.014	0.065	-+	0.163
GPAM	10	rs2255141	G	-0.014	0.036	0.710	0.027	0.017	0.121	0.020	0.015	0.204	-+	0.303
JMJD1C	10	rs10761731	A	0.012	0.032	0.700	0.018	0.016	0.240	0.017	0.014	0.240	++	0.867
AMPD3	11	rs2923084	A	-0.007	0.043	0.870	-0.037	0.020	0.061	-0.032	0.018	0.081	--	0.527
APOA1	11	rs964184	C	-0.240	0.049	1.20×10^{-06}	-0.291	0.023	2.10×10^{-37}	-0.282	0.021	9.80×10^{-42}	--	0.346
FADS1-2-3	11	rs174546	C	-0.026	0.035	0.460	-0.064	0.016	6.30×10^{-05}	-0.057	0.015	7.92×10^{-05}	--	0.323
LRP4	11	rs3136441	T	0.019	0.049	0.700	0.072	0.022	0.001	0.063	0.020	0.002	++	0.324
SPTY2D1	11	rs10128711	C	-0.022	0.036	0.540	0.035	0.017	0.042	0.025	0.015	0.109	-+	0.152
ST3GAL4	11	rs11220462	G	0.041	0.048	0.390	-0.010	0.022	0.670	-0.001	0.020	0.954	+-	0.334
UBASH3B	11	rs7941030	T	0.025	0.033	0.450	-0.008	0.016	0.610	-0.002	0.014	0.905	+-	0.368
BRAP	12	rs11065987	A	-0.020	0.032	0.540	-0.029	0.016	0.059	-0.027	0.014	0.057	--	0.801
HNF1A	12	rs1169288	A	-0.085	0.034	0.012	-0.009	0.017	0.600	-0.024	0.015	0.112	--	0.046
LRP1	12	rs11613352	C	0.078	0.038	0.039	0.041	0.018	0.021	0.048	0.016	0.003	++	0.379
MVK	12	rs7134594	T	0.014	0.032	0.660	-0.002	0.015	0.880	0.001	0.014	0.948	+-	0.651
PDE3A	12	rs7134375	C	-0.015	0.032	0.650	0.022	0.015	0.147	0.015	0.014	0.259	-+	0.295
SBNO1	12	rs4759375	C	-0.136	0.068	0.046	0.011	0.032	0.740	-0.016	0.029	0.589	-+	0.050
SCARB1	12	rs838880	T	-0.026	0.035	0.470	0.013	0.017	0.460	0.006	0.015	0.716	-+	0.316

				Within Adolescent			Within Adult			Adolescent + Adult				
Locus	Chr	SNP	Ref Allele	Beta	SE	P-value	Beta	SE	P-value	Beta	SE	P-value	Direction	Heterogeneity p-value
ZNF664	12	rs4765127	G	0.024	0.034	0.480	0.067	0.016	3.10×10^{-05}	0.059	0.015	4.32×10^{-05}	++	0.253
NYNRIN	14	rs8017377	G	-0.028	0.032	0.390	0.008	0.015	0.600	0.002	0.014	0.911	-+	0.308
CAPN3	15	rs2412710	G	-0.053	0.120	0.660	-0.071	0.058	0.210	-0.068	0.052	0.196	--	0.893
FRMD5	15	rs2929282	A	-0.172	0.080	0.033	-0.044	0.039	0.260	-0.069	0.035	0.050	--	0.150
LACTB	15	rs2652834	G	-0.062	0.041	0.134	0.004	0.020	0.830	-0.009	0.018	0.629	-+	0.148
LIPC	15	rs1532085	G	-0.041	0.033	0.210	-0.062	0.016	6.30×10^{-05}	-0.058	0.014	5.61×10^{-05}	--	0.567
CETP	16	rs3764261	C	0.046	0.034	0.170	0.037	0.016	0.024	0.039	0.015	0.008	++	0.811
CMIP	16	rs2925979	C	0.023	0.036	0.530	-0.022	0.017	0.190	-0.014	0.015	0.370	+-	0.258
CTF1	16	rs11649653	C	0.028	0.034	0.420	0.027	0.016	0.090	0.027	0.015	0.060	++	0.979
HPR	16	rs2000999	G	0.061	0.041	0.139	-0.009	0.020	0.640	0.005	0.018	0.804	+-	0.125
LCAT	16	rs16942887	G	-0.082	0.052	0.112	0.039	0.024	0.101	0.018	0.022	0.415	-+	0.035
ABCA8	17	rs4148008	C	0.031	0.035	0.370	0.008	0.017	0.640	0.012	0.015	0.418	++	0.554
OSBPL7	17	rs7206971	G	0.017	0.032	0.590	0.022	0.015	0.145	0.021	0.014	0.120	++	0.888
PGS1	17	rs4129767	G	-0.008	0.032	0.790	-0.006	0.015	0.700	-0.006	0.014	0.640	--	0.955
STARD3	17	rs11869286	C	-0.021	0.033	0.530	-0.014	0.016	0.380	-0.015	0.014	0.287	--	0.849
LIPG	18	rs7241918	T	0.027	0.041	0.510	-0.021	0.020	0.280	-0.012	0.018	0.513	+-	0.293
MC4R	18	rs12967135	G	-0.017	0.039	0.660	-0.047	0.018	0.008	-0.042	0.016	0.011	--	0.485
ANGPTL4	19	rs7255436	A	0.027	0.032	0.400	-0.046	0.015	0.003	-0.033	0.014	0.016	+-	0.039
APOE	19	rs439401	C	0.112	0.034	0.0008	0.076	0.016	1.90×10^{-06}	0.083	0.015	1.19×10^{-08}	++	0.338
APOE	19	rs4420638	A	-0.094	0.047	0.045	-0.093	0.022	1.50×10^{-05}	-0.093	0.020	2.92×10^{-06}	--	0.985
CILP2	19	rs10401969	T	0.153	0.060	0.011	0.055	0.029	0.056	0.074	0.026	0.005	++	0.141
FLJ36070	19	rs492602	G	-0.002	0.032	0.950	0.026	0.015	0.087	0.021	0.014	0.123	-+	0.428
LDLR	19	rs6511720	G	-0.060	0.050	0.230	-0.013	0.024	0.590	-0.022	0.022	0.314	--	0.397
LILRA3	19	rs386000	G	0.011	0.039	0.770	0.035	0.019	0.062	0.030	0.017	0.075	++	0.580
LOC55908	19	rs737337	T	0.016	0.062	0.800	-0.010	0.029	0.720	-0.005	0.026	0.839	+-	0.704
ERGIC3	20	rs2277862	C	-0.052	0.044	0.240	0.002	0.022	0.920	-0.009	0.020	0.655	-+	0.272
MAFB	20	rs2902940	A	-0.018	0.034	0.590	0.033	0.016	0.042	0.024	0.015	0.101	-+	0.175
PLTP	20	rs6065906	T	-0.022	0.043	0.600	-0.055	0.019	0.005	-0.050	0.017	0.004	--	0.483
TOP1	20	rs6029526	T	-0.015	0.032	0.640	-0.012	0.015	0.420	-0.013	0.014	0.356	--	0.932
PLA2G6	22	rs5756931	T	0.037	0.033	0.260	0.000	0.016	0.990	0.007	0.014	0.625	0	0.313
UBE2L3	22	rs181362	C	-0.041	0.041	0.320	0.024	0.020	0.220	0.012	0.018	0.522	-+	0.154

Numbers in ‘Beta’ and ‘SE’ columns are in standard deviation (SD) unit. The SD unit for adolescents and adults are 0.183 and 0.216 respectively.
