# Supplementary material for: Evidence of Differential Allelic Effects between Adolescents and Adults for Plasma High-Density Lipoprotein
Source: PLoS One. 2012 Apr 18;7(4):e35605. doi: 10.1371/journal.pone.0035605 (PMC3329456; doi:10.1371/journal.pone.0035605)
Supplement: Table S8 — The locus/phenotype combinations of EN-others heterogeneity tests reported Table 2. (PDF) [file pone.0035605.s012.pdf]

Table S8. The locus/phenotype combinations of EN-others heterogeneity test reported Table2.

						EN			Others			EN+Others				
**Trait**	**Locus**	**Chr**	**SNP**	**Allele/ MAF**	**Ref Allele**	**Beta**	**SE**	**P-value**	**Beta**	**SE**	**P-value**	**Beta**	**SE**	**P-value**	**Direction**	**Het p-value**
HDL	PABPC4	1	rs4660293	A/G/0.25	A	0.053	0.059	0.370	0.064	0.020	0.0011	0.063	0.019	0.0009	++	0.860
	ZNF664	12	rs4765127	G/T/0.34	G	–0.133	0.052	0.010	–0.079	0.017	3.80×10^{-06}	–0.084	0.016	1.87×10^{-07}	--	0.324
	MVK	12	rs7134594	T/C/0.47	T	0.081	0.048	0.091	0.000	0.016	1.00	0.008	0.015	0.059	0	0.109
	CETP	16	rs3764261	C/A/0.32	C	–0.307	0.052	3.90×10^{-09}	–0.236	0.017	8.00×10^{-43}	–0.243	0.016	4.69×10^{-51}	--	0.194
	MC4R	18	rs12967135	G/A/0.24	G	–0.045	0.059	0.450	0.040	0.019	0.034	0.032	0.018	0.077	+-	0.170
LDL	LDLR	19	rs6511720	G/T/0.11	G	0.212	0.084	0.0121	0.173	0.026	2.40×10^{-11}	0.176	0.025	1.23×10^{-12}	++	0.657

“Allele/MAF” listed are: major allele, minor allele frequency (MAF). Numbers in “Beta” columns are in SD units, modelled as additive effect of the reference allele.
